# PI5P4Kα supports prostate cancer metabolism and exposes a survival vulnerability during androgen receptor inhibition

**DOI:** 10.1126/sciadv.ade8641

**Published:** 2023-02-01

**Authors:** Joanna Triscott, Matthias Reist, Lukas Küng, Francielle C. Moselle, Marika Lehner, John Gallon, Archna Ravi, Gurpreet K. Arora, Simone de Brot, Mark Lundquist, Hector Gallart-Ayala, Julijana Ivanisevic, Salvatore Piscuoglio, Lewis C. Cantley, Brooke M. Emerling, Mark A. Rubin

**Affiliations:** ^1^ Department for BioMedical Research, University of Bern, Bern 3008, Switzerland.; ^2^ Institute of Biosciences, São Paulo State University, São Paulo, Brazil.; ^3^ Visceral Surgery and Precision Medicine Research Laboratory, Department of Biomedicine, University of Basel, Basel, Switzerland.; ^4^ Cell and Molecular Biology of Cancer Program, Sanford Burnham Prebys, La Jolla, CA 92037, USA.; ^5^ COMPATH, Institute of Animal Pathology, University of Bern, Bern, Switzerland.; ^6^ Meyer Cancer Center, Weill Cornell Medicine and New York Presbyterian Hospital, New York, NY 10065, USA.; ^7^ Metabolomics Platform, Faculty of Biology and Medicine, University of Lausanne, Lausanne, Switzerland.; ^8^ Dana-Farber Cancer Institute, Harvard Medical School, Boston, MA 02215, USA.; ^9^ Bern Center for Precision Medicine, University of Bern and Inselspital, Bern 3008, Switzerland.

## Abstract

Phosphatidylinositol (PI)regulating enzymes are frequently altered in cancer and have become a focus for drug development. Here, we explore the phosphatidylinositol-5-phosphate 4-kinases (PI5P4K), a family of lipid kinases that regulate pools of intracellular PI, and demonstrate that the PI5P4Kα isoform influences androgen receptor (AR) signaling, which supports prostate cancer (PCa) cell survival. The regulation of PI becomes increasingly important in the setting of metabolic stress adaptation of PCa during androgen deprivation (AD), as we show that AD influences PI abundance and enhances intracellular pools of PI-4,5-P_2_. We suggest that this PI5P4Kα-AR relationship is mitigated through mTORC1 dysregulation and show that PI5P4Kα colocalizes to the lysosome, the intracellular site of mTORC1 complex activation. Notably, this relationship becomes prominent in mouse prostate tissue following surgical castration. Finally, multiple PCa cell models demonstrate marked survival vulnerability following stable PI5P4Kα inhibition. These results nominate PI5P4Kα as a target to disrupt PCa metabolic adaptation to castrate resistance.

## Teaser

An understanding of the metabolic function of PI5P4Kα shows potential for targeting it in prostate cancer.

## INTRODUCTION

**Fig. 1.**
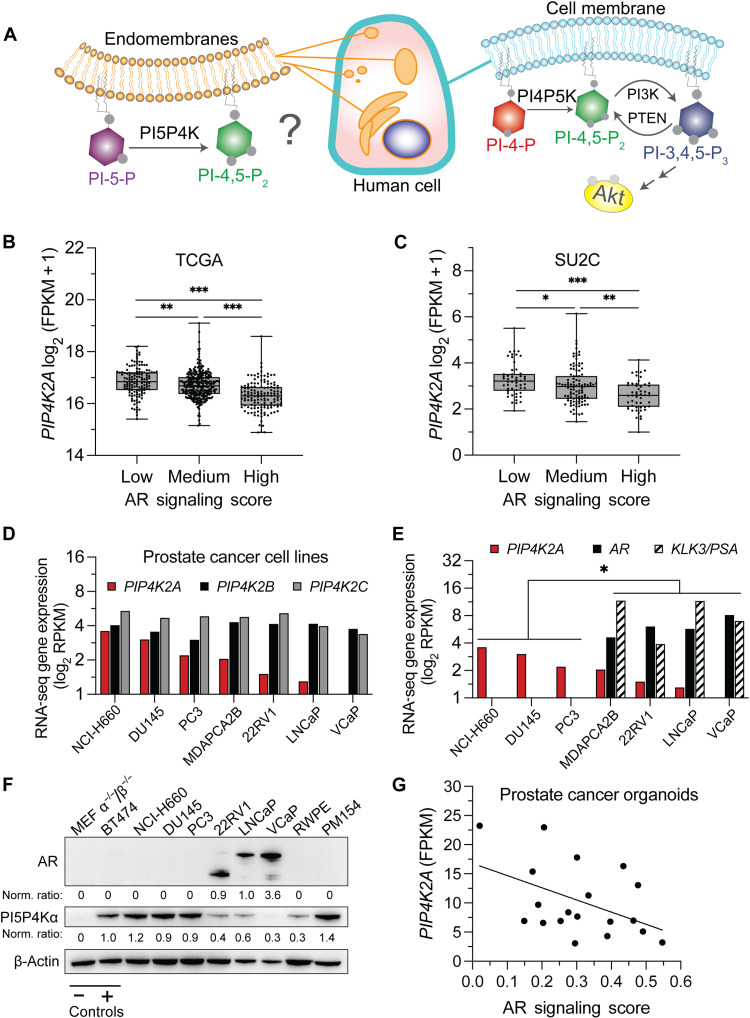
*PIP4K2A* expression correlates with low androgen receptor signaling. (**A**) Schematic comparing known kinase activity of noncanonical (left) and canonical (right) phosphoinositide kinase pathways. PI5P4K generates PI-4,5-P_2_ from a PI-5-P lipid instead of PI-4-P. PI5P4K functions at internal organelles of the endomembrane system as opposed to the exterior cell membrane–like canonical pathway. The importance of PI3K signaling has been established in prostate cancer (PCa), but the question remains how PI5P4K affects prostate biology and androgen receptor (AR) signaling. (**B**) Analysis of *PIP4K2A* transcript expression shows inverse correlation with AR signature score (*28*) on available transcript data from The Cancer Genome Atlas (PRAD-TCGA; *N* = 333, *P* = 4.9 × 10^−16^) and (**C**) Stand Up to Cancer datasets (SU2C; *N* = 149, *P* = 5.1 × 10^−6^). (**D**) RNA sequencing (RNA-seq) transcript abundance for established PCa cells lines for *PIP4K2A*, *PIP4K2B*, and *PIP4K2C* isoforms. (**E**) Highest levels of *PIP4K2A* transcript expression occurs in *AR* and *KLK3* negative models (ARpos mean, 1.127; ARneg mean, 2.941; *t* test, *P* = 0.048). (**F**) Western blot analysis with mouse embryonic fibroblast (MEF) double knockout (negative control) and BT474 (positive control) confirms protein expression of PI5P4Kα in PCa cell lines. (**G**) *PIP4K2A* expression further characterized in a panel of human PCa organoids using RNA-seq (*R*^2^ = 0.2146, *P* = 4.5 × 10^−2^). *t* test values: *P < 0.05; **P < 0.01; and ***P < 0.001.

In 1997, the Cantley laboratory found a kinetic function of an unexplored family of lipid kinases called phosphatidylinositol-5-phosphate 4-kinases (PI5P4Ks) (*1*). PI5P4K acts by phosphorylating the minor lipid phosphatidylinositol-5-phosphate (PI-5-P) at the 4 position of the inositol ring to generate phosphatidylinositol-4,5-bisphosphate (PI-4,5-P_2_; PIP_2_) (Fig. 1A). Humans and mice have three distinct genes—*PIP4K2A*, *PIP4K2B*, and *PIP4K2C*—encoding enzymes called PI5P4Kα, PI5P4Kβ, and PI5P4Kγ, respectively. Knockout mice have been generated for all three isoforms and are viable with normal life span and subtle phenotypes (*2*–*4*). Although mice deficient in the two most active PI5P4Ks (*Pip4k2a^–/–^Pip4k2b^–/–^*) develop into normal embryos, they fail to survive longer than 12 hours after birth (*2*). Furthermore, a strong connection of the PI5P4K isoforms has been established in cancers, as they are dysregulated in several cancer subtypes, including breast, glioblastomas, acute myeloid leukemia (AML), and sarcomas (*2*, *5*–*8*). In previous work, knocking down the PI5P4Kα and PI5P4Kβ isoforms in *TP53-*mutant breast cancer cells inhibits cell viability, and that deletion of the enzymes in mice suppresses tumor formation with *Trp53* deletion (*2*). Moreover, our recent studies demonstrated the requirement of both the PI5P4Kα and PI5P4Kβ isoforms for sarcoma tumor initiation and maintenance (*9*).

Recently, the roles of the PI5P4K isoforms in controlling cellular metabolism have been found (*9*, *10*), and we posit that targeting PI5P4K could expose critical vulnerabilities in cancer. While most of the cellular PI-4,5-P_2_ is localized at the plasma membrane and generated by the canonical pathway in which PI-4-P is phosphorylated at the 5 position of the inositol ring (*11*, *12*), intracellular PI-4,5-P_2_ can be synthesized by an alternative type 2 pathway in which PI5P4Ks convert PI-5-P to PI-4,5-P_2_ at the membranes of endosomes, lysosomes, Golgi apparatus, endoplasmic reticulum (ER), nucleus, and peroxisomes (*13*, *14*). Notably, while PI5P4K-generated peroxisomal PI-4,5-P_2_ is necessary for the peroxisome-lysosome membrane contacts that facilitate cholesterol transport between organelles (*15*), the PI-4,5-P_2_ lipid has many other functions that could be important. This includes the control of the activity of membrane transport proteins that allow influx and efflux of metabolites from lysosomes, as well as roles in the localization of signaling complexes through lipid-protein binding interactions (*16*). While the precise spatial and temporal control of PI-4,5-P_2_ at intracellular membranes is important for cellular stress response, the influence of PI5P4K and its relationship with many key cancer pathways have yet to be explored.

The PI5P4Ks are lesser-known members of a network of enzymes that regulate phosphorylated phosphatidylinositol (PI). These kinases and phosphatases that control the addition or removal of phosphates on the inositol ring of PI has been established as a cornerstone of multiple biological processes and have a high frequency of alteration across most cancer types (*17*, *18*). The most well-known class is the phosphoinositide 3-kinases (type 1 PIP kinase pathway; PI3Ks), which modulate critical cellular functions for development, immunity, and tumorigenesis. The PI3K pathway also has a major role in regulating metabolism. It conducts downstream signals from the stimulated insulin receptor through phosphorylation of AKT serine/threonine kinase to control glucose homeostasis. To date, there have been more than 700 clinical trials involving PI3K/AKT targeted molecules, with an estimated 334 of these trials listed as ongoing or currently planned (ClinicalTrials.gov). These involve a spectrum of cancer types including acute lymphocytic leukemia, AML, glioma, melanoma, colorectal, lung, breast, and prostate cancer (PCa) (*19*). There is tremendous progress in the development of PI3K inhibitors for the treatment of cancer; however, as with many targeted therapies, full efficacy may be stunted by dose-limiting toxicities and the acquisition of resistance. By exploring PI5P4K, we hope to understand the broader network of phosphoinositide regulators with the goal of exposing metabolic cancer vulnerabilities that support clinically used therapeutics.

The functional role of PI5P4K can vary markedly between tissue and cell types. In this study, we focused our efforts solely on PCa and, to our knowledge, are the first to characterize PI5P4K in this disease. An estimated one of seven men will develop PCa by the age of 60. While treatment strategies for localized disease can be curative, 10 to 20% of PCa will progress to advanced castrate-resistant disease [castrate-resistant PCa (CRPC)] within 5 years (*20*). The prostate is a specialized reproductive gland that is particularly susceptible to cancer development. Normal prostate cell growth and development are heavily dependent on androgen receptor (AR) signaling. Androgens strongly influence the metabolic state of prostate cells to favor sustained cellular growth (*21*). Reinforcing this growth, the PI3K pathway has the highest frequency of alteration in both primary and metastatic PCa (*22*). A direct connection has been established between the PI3K-AKT-mTOR (mammalian target of rapamycin) and AR signaling axis in the prostate. Pathway deregulation resulting from loss of phosphatase and tensin homolog (PTEN) is associated with the down-regulation of AR target genes (*23*–*25*). While foundational studies support the importance of phosphoinositide signaling in AR regulation, the entire picture of the intricate cross-talk between these systems remains unclear.

There are a growing number of inhibitors being tested in combination with androgen-targeted therapies in clinical trials (*26*, *27*) but still almost nothing is known about the potential cross-talk of the greater phosphoinositide kinase network. Here, we suggest an interplay of PI5P4K in AR signaling and lay the foundation for phenotypic understanding of the role of PI5P4Kα in prostate metabolism.

## RESULTS

### PIP4K2A inversely correlates with AR signaling in PCa

To establish the degree and frequency of *PIP4K2* in PCa, we queried two publicly available patient datasets for patterns of gene expression. *PIP4K2A*, *PIP4K2B*, and *PIP4K2C* transcript expression was detected in clinically localized [The Cancer Genome Atlas (TCGA)-Prostate Adenocarcinoma (PRAD), *N* = 498] and advanced, metastatic PCa [Stand Up 2 Cancer-Prostate Cancer Foundation (PCF) (SU2C, *N* = 209)] datasets. *PIP4K2A* and *PIP4K2B* demonstrate similar expression patterns, while the *PIP4K2C* transcript was expressed differentially from the other two isoforms (fig. S1, A and B). Previous work associated *PIP4K2B* with tumor protein p53 (*TP53)* mutant breast cancer (*2*). *TP53* and *PTEN* are among the most altered genes in CRPC; however, we found that none of the *PIP4K2* isoforms associate with *TP53* nor *PTEN* mutation in CRPC (fig. S1, C and D). In addition, the genes coding for the *PIP4K2* isoforms are not commonly mutated in either TCGA or SU2C datasets.

To determine whether PI5P4K relates to other know drivers of PCa, we tested whether there is a relationship between *PIP4K2A* and AR signaling. A previously established AR signaling score, which includes gene signature changes resulting from pathway stimulation, can be used as a metric of AR activity in tumor material (table S1) (*28*). AR signaling generally will increase with up-regulation of AR in early PCa development then diminish with disease progression into AR-independent CRPC. In PCa patient datasets, the highest levels of *PIP4K2A* correlated with lower AR signaling in both TCGA (PRAD, *P* = 4.9 × 10^−16^, *N* = 498) and SU2C datasets (SU2C, *P* = 5.1 × 10^−6^, *N* = 209) (Fig. 1, B and C). *PIP4K2B* and *PIP4K2C* isoforms showed no correlation to the AR pathway (fig. S1E). These transcript observations prompted us to optimize immunohistochemical (IHC) reagents to confirm protein-level expression in patient tissue. PI5P4Kα staining of human clinical samples revealed high cytoplasmic positivity in areas of basal cell hyperplasia and inflammation (fig. S2, A and B). Antibody specificity was further supported by comparing serial section localization of an RNA–in situ hybridization (ISH) probe for *PIP4K2A* (fig. S2, C and D). Together, these data show that PI5P4Kα is present in human PCa tissue and correlates with decreasing AR signaling.

Next, we profiled a panel of PCa cell lines for transcript expression of the *PIP4K2* isoforms (Fig. 1D). While the transcript abundance of *PIP4K2B* and *PIP4K2C* did not vary between cell lines, *PIP4K2A*, the most catalytically active isoform (*29*, *30*), shows a marked range of expression between cell lines with NCI-H660 (neuroendocrine PCa model) and VCaP (AR responsive) being the highest and lowest, respectively. An enrichment for *PIP4K2A* occurs in AR-negative cells that model CRPC (i.e., PC3, DU145, and NCI-H660) compared to AR-responsive cells that retain AR and *KLK3* transcript expression (i.e., VCaP, LNCaP, 22RV1, and MDAPCA2B) (Fig. 1E). In addition, the *PIP4K2A* relative transcript levels are paralleled on the protein level (Fig. 1F). Furthermore, we queried the expression of *PIP4K2* isoforms in a panel of PCa patient-derived organoids. This panel includes rare patient-derived cell models that recapitulate the disease of PCa to a high standard of in vitro culture technology (tables S2 and S3) (*31*–*33*). Here, an inverse correlation of *PIP4K2A* is again found in the analysis of bulk RNA sequencing (RNA-seq) data (Fig. 1G and fig. S3A). Collectively, cell lines and organoid models demonstrate that *PIP4K2A* expression levels are the highest in PCa models with diminished AR signaling.

### PIP4K2A has a functional relationship with AR

**Fig. 2.**
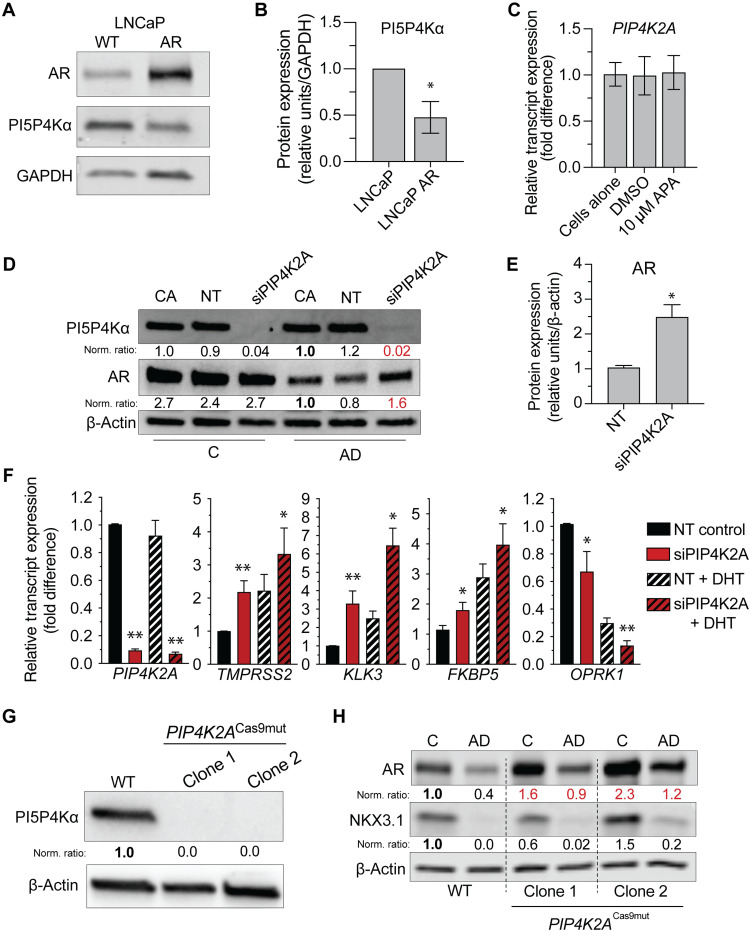
*PIP4K2A* has a functionally inverse relationship with *AR* in PCa cell models. (**A**) Stable overexpression of AR in LNCaP model corresponds to decrease in PI5P4Kα protein by Western blot, which is quantified with (**B**) protein densitometry (*N* = 3, 0.48-fold of control, *P =* 6.0 × 10^−3^). GAPDH, glyceraldehyde-3-phosphate dehydrogenase. (**C**) Following a 48-hour treatment of AR inhibitor, 10 μM APA does not alter *PIP4K2A* transcript or (**D**) PI5P4Kα protein expression (CA, cells alone; NT, nontargeting control). (**E**) Protein densitometry of AR following *siPIP4K2A* knockdown in androgen depletion (AD) (*n* = 3, 2.49-fold of control, *P =* 2.9 × 10^−2^). (**F**) AR signature transcript expression is boosted following 48-hour siRNA transfection of LNCaP cells and 18-hour DHT stimulation in AD. (**G**) Western blot of stable *PIP4K2A*-targeted LNCaP clones using CRISPR-Cas9 lacking protein expression and (**H**) shows up-regulated AR relative to wild-type (WT) control clones and mutant clones (*PIP4K2A*^Cas9mut^). Immunoblot quantification values shown following housekeeper and control normalization (relative to bolded sample). Red values highlight experimental change. *t* test values: *P < 0.05; **P < 0.01.

To explore the correlation between *PIP4K2A* and AR, we modulated PCa AR signaling in vitro. We did this using the LNCaP and LNCaP-AR models of human adenocarcinoma, as these cells retain AR pathway activity with LNCaP-AR modified to overexpress AR (*34*). We found LNCaP-AR to have only 0.48-fold the PI5P4Kα protein relative to wild-type LNCaP (*N* = 3, *P =* 6.0 × 10^−3^; Fig. 2, A and B). Compared to AR-negative CRPC models (i.e., PC3), wild-type LNCaP has relatively low PI5P4Kα that is reduced further with the overexpression of AR. Conversely, the acute inhibition of AR with apalutamide (APA) does not affect *PIP4K2A* transcript (Fig. 2C). This suggests that *PIP4K2A* is not a direct target of AR transcription factor activity, and the PI5P4Kα-AR correlation is mechanistically indirect.

To investigate whether inhibition of PI5P4Kα functionally affects the AR activity, we further examined the outcome of its in vitro down-regulation. Small interfering RNA (siRNA) targeting of *PIP4K2A* (siPIP4K2A) in LNCaP cells demonstrated minimal change to AR or AR target genes in complete medium conditions; however, when cultured in androgen deprivation (AD) conditions, *siPIP4K2A* treatment resulted in an up-regulation of AR signature transcripts and AR protein (Fig. 2, D to F, and fig. S3, B and C). Depleted of hormones and large hydrophobic molecules, the AD medium contains charcoal-stripped (C/S) serum that lacks dihydrotestosterone (DHT) and other androgen substrate derivatives known to activate AR (*35*). This boost in AR transcript is comparable to levels following control DHT stimulation, and the combination of siPIP4K2A with DHT resulted in an additive boost of AR signature genes (i.e., *TMPRSS2*, *KLK3*, and *FKBP5* increase; *OPRK1* decrease; Fig. 2F). CRISPR-Cas9 tools and *PIP4K2A-*targeted small guide RNAs allowed production of LNCaP clones lacking PI5P4Kα protein (Fig. 2G). Similar to the acute siRNA treatments, *PIP4K2A^Cas9mut^* LNCaP, expanded from rare single-cell clones, had up-regulated AR expression (Fig. 2H). These data provide evidence that PI5P4Kα has a functionally dynamic interplay with AR.

### PIP4K2A-AR relationship is nonredundant to PI3K inhibition

**Fig. 3.**
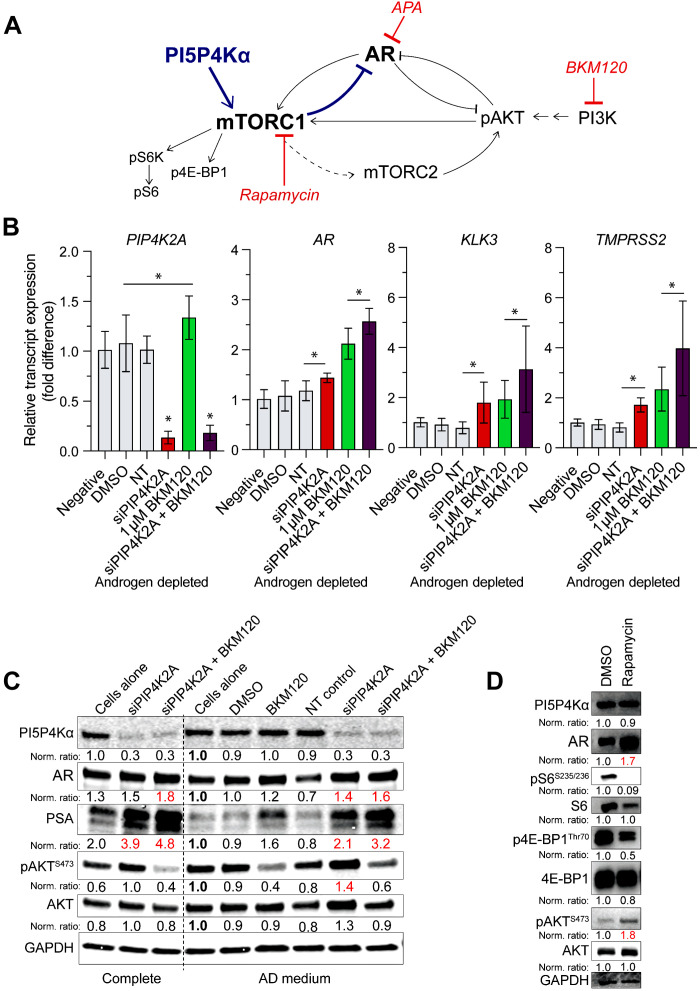
PI5P4Kα is nonredundant to the PI3K pathway. (**A**) A relationship has been established between AR and the PI3K (type 1) pathway. Graphical summary of suggested feedback of AR when PI3K/AKT or mTORC1 is inhibited. (**B**) LNCaP cells in androgen deprivation (AD) were treated with siRNA targeting *PIP4K2A* (siPIP4K2A) for 48 hours and/or 10 μM BKM120 for 24 hours. Changes in *AR* transcript expression and AR signature genes (*KLK3* and *TMPRSS2*) by quantitative real-time polymerase chain reaction (qRT-PCR) are represented. (**C**) Western blot analysis also compares protein-level changes following *siPIP4K2A* and/or 10 μM BKM120. (**D**) Signaling changes from siPIP4K2A treatment of LNCaP appear to phenocopy treatment with rapamycin. Phospho-target changes are represented using Western blot comparison of 1 nM rapamycin compared to dimethyl sulfoxide (DMSO) control for 24 hours. Immunoblot quantification values shown following housekeeper and control normalization (relative bolded sample). Red values highlight experimental change. *t* test values: *P < 0.05.

PI5P4Kα is not the only metabolic factor with which AR dynamically interacts. Inverse relationships with AR have also been shown following the inhibition of mTOR complex 1 (mTORC1) (*36*, *37*) as well as through targeting PI3K, a well-known PI kinase (Fig. 3A) (*23*, *38*). Therefore, we questioned whether the boost in AR activity resulting from *PIP4K2A* depletion is mitigated through the PI3K-AR relationship. Buparlisib (BKM120) used at 1 μM concentration was used to compare PI3K inhibition to *PIP4K2A* knockdown in LNCaP cells. As expected, siPIP4K2A and BKM120 treatments as single agents caused a boost to *AR* and AR target genes. Unexpectedly, combination treatment of siPIP4K2A with 24 hours of BKM120 showed an additive boost in AR target genes even in AD conditions (Fig. 3B). This boost was further confirmed on protein level in both complete medium and AD conditions; however, siPIP4K2A differed from BKM120, as it produced an up-regulation of phospho-AKT^S473^ (Fig. 3C). Up-regulation of AR and feedback that increases phospho-AKT^S473^ is a phenotype of mTORC1 inhibition (*36*, *39*). Figure 3D demonstrates this following a 24-hour treatment of LNCaP cells with 1 nM rapamycin. Down-regulation of mTORC1 is exemplified by a reduction in targets phospho-S6^S235/236^ and phospho–4E-BP1^Thr70^, whereas AR protein and phospho-AKT^S473^ are elevated. Levels of PI5P4Kα remain the same following rapamycin treatment (Fig. 3D). Concerning AR signaling, these findings suggest that *PIP4K2A* depletion is not redundant with PI3K inhibition.

### PI5P4Kα influences AR signaling through dysregulation of mTORC1

**Fig. 4.**
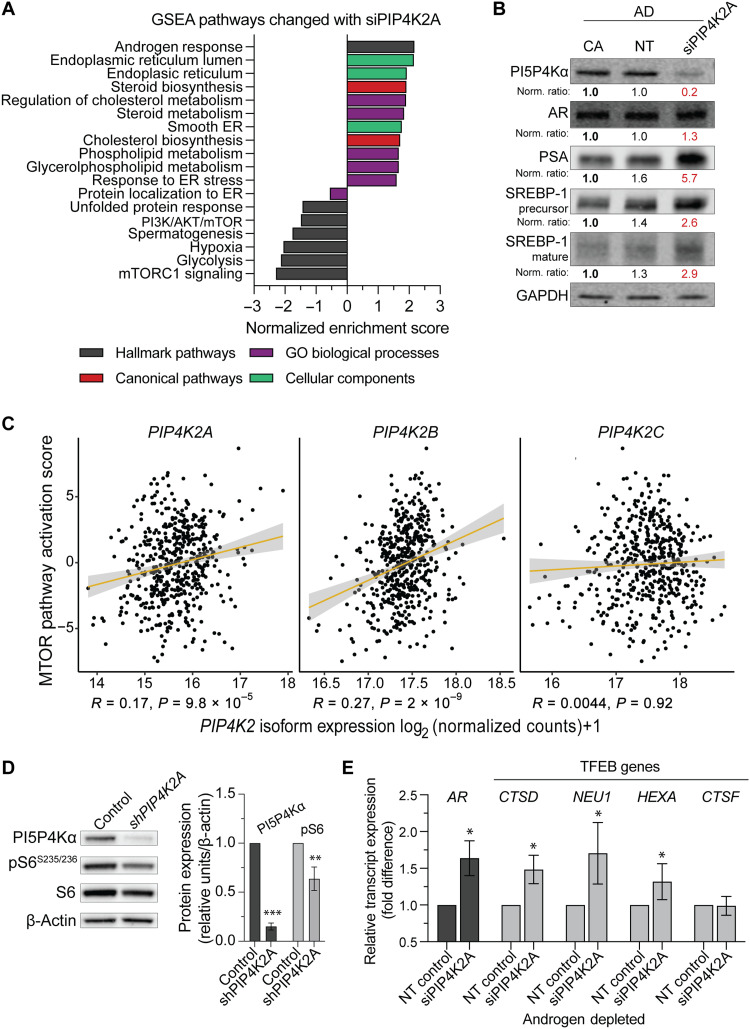
PI5P4Kα affects mTORC1 signaling. (**A**) Whole-exome transcript expression was measured with bulk RNA-seq. Statistically significant differential GSEA pathways between nontargeting siRNA control (NT) and siRNA specific for *PIP4K2A* (siPIP4K2A) treatments are represented. Pathways shown all with *P*_adj_ significant *P* < 0.05. (**B**) Western blotting confirms changes in AR pathway targets following a knockdown in PI5P4Kα protein expression. (**C**) Further analysis of patient data from TCGA (PRAD-TCGA) shows a positive association with *PIP4K2A* (*R* = 0.17, *P =* 9.8 × 10^−5^) and an mTOR activation score (*17*). In addition, *PIP4K2B* (*R* = 0.27, *P =* 2 × 10^−9^) but not *PIP4K2C* (*R* = 0.0044, *P* = 0.92). (**D**) LNCaP cells infected with pLKO.1 lentiviral *PIP4K2A* shRNAs (shPIP4K2A) show reduced phospho-S6^235/236^ 48 hours after GFP sorting. Immunoblot quantification values shown following housekeeper and control normalization (relative bolded sample). Red values highlight experimental change. (**E**) LNCaP cells in androgen deprivation (AD) were treated with si*PIP4K2A* for 48 hours. Changes of *AR* transcript expression and TFEB signature genes (*CTSD*, *NEU1*, *HEXA*, and *CTSF*) by qRT-PCR are represented. Data representative of three independent experiments, mean ± SEM. *t* test values: *P < 0.05; **P < 0.01; and ***P < 0.001.

Next, we sought to understand which molecular pathways are influenced by targeting PI5P4Kα. We conducted transcriptomic sequencing of LNCaP cells following *PIP4K2A* knockdown in AD conditions. There were few single transcripts changed between nontargeting (NT) and siPIP4K2A based on a fold change cut of 50% (fig. S4A). However, many biological processes involving fundamental metabolism pathways were affected by *PIP4K2A* depletion, as exemplified by selected gene set enrichment analysis (GSEA) pathway enrichment from whole-exome RNA-seq (Fig. 4A). Notably, androgen response genes were of the highest up-regulation with *PIP4K2A* knockdown. Statistically significant enrichment scores were also observed for pathways involving ER stress, steroid biosynthesis, cholesterol metabolism, phospholipid metabolism, hypoxia, and glycolysis. AR and lipid pathways were further implicated in *PIP4K2A* regulation on a companion Western blot showing up-regulation of AR, prostate-specific antigen, and sterol regulatory element–binding protein 1 (Fig. 4B). A −2.3 normalized enrichment score (*P*_adj_ = 0.012) for mTORC1 signaling stood out as a Hallmark pathway as one of the most reduced in RNA-seq (Fig. 4A and fig. S4B). Transcriptomic analysis confirmed that depletion of *PIP4K2A* boosts AR signature genes and dysregulates essential pathways such as lipid metabolism and mTORC1 signaling.

Molecular alterations to pathways converging on mTORCs are common across most cancer types (*17*). Zhang *et al*. (*17*) previously defined a score for mTOR using multicancer TCGA analysis of proteomic and transcriptomic data. In the present study, the score for mTOR pathway activation had a positive correlation with *PIP4K2A* expression in PCa cases (TCGA PRAD, *R* = 0.17, *P* = 9.8 × 10^−5^). Furthermore, *PIP4K2B* was also correlated (*R* = 0.27, *P* = 2 × 10^−9^) but with a narrow expression profile distribution. No correlation was found for *PIP4K2C* with mTOR pathway activation (*R* = 0.0044, *P* = 0.92; Fig. 4C). This relationship was validated using RNA-seq data from *PIP4K2A* knockdown in LNCaP cells (fig. S4C), as well as with Western blot measurement of mTORC1 target phospho-S6 ribosomal protein (phospho-S6^235/236^) following stable lentiviral infection of a short hairpin RNA (shRNA) targeting *PIP4K2A* (shPIP4K2A) for 72 hours (0.64-fold of control; *t* test, *P* = 0.006; Fig. 4D). Note that under conditions of nutrient stress, catabolic pathways controlled by mTORC1 are activated. For example, the loss of phosphorylation of the mTORC1 target, transcription factor EB (TFEB), allows it to translocate to the nucleus and activate transcription programs for lysosome biogenesis and stress metabolism. TFEB transcription activation is a biomarker of loss of mTORC1 anabolic activity, and we observed an up-regulation of three of four tested TFEB target genes (*CTSD*, *NEU1*, *HEXA*, and *CTSF*) following siPIP4K2A depletion in LNCaP cells in AD (Fig. 4E). Activation of TFEB via PI5P4K-mediated mTORC1 suppression is established for both genetic knockout and kinase drug inhibitor studies (*10*, *40*). These data imply an association of PI5P4Kα with mTORC1 signaling, a key pathway used in PCa progression.

### Androgen deprivation stress enriches for PI lipids

**Fig. 5.**
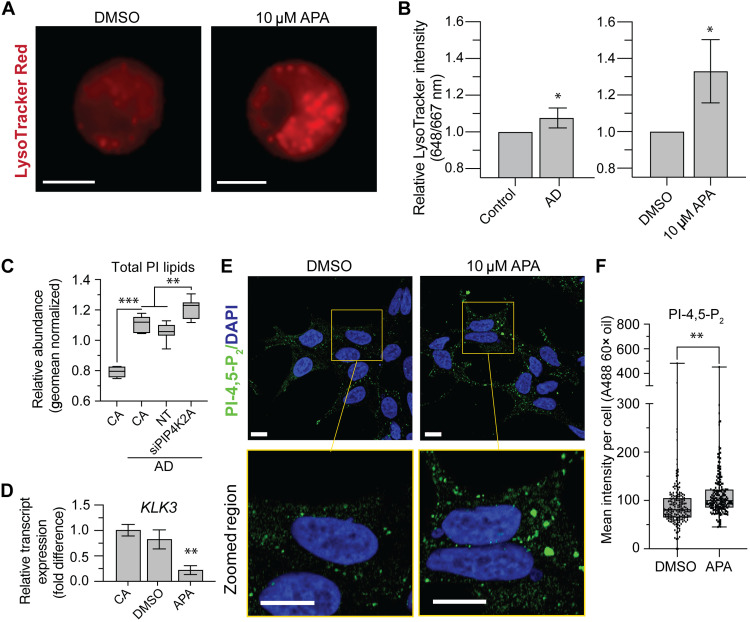
AR inhibition dysregulates PI signaling at the endolysosome. (**A**) LysoTracker Red IF staining of LNCaP cells treated with 10 μM APA or DMSO for 48 hours. Scale bars, 10 μm. (**B**) Relative quantification of LysoTracker Red IF intensity in cell populations following 48 hours androgen deprivation (AD) or APA treatment. Data representative of three independent experiments. (**C**) LNCaP cultured in complete RPMI 1640 + 5% FBS compared to AD conditions. Total PI lipids were measured in LNCaP cells transfected with siRNA targeting *PIP4K2A* (siPIP4K2A) for 24 hours and then switched into AD conditions for an additional 48 hours. Relative PI lipid content was measured using lipidomic liquid chromatography–tandem mass spectrometry (LC-MS/MS) methodology. CA, cell alone in complete medium unless indicated with AD; NT, nontargeting siRNA control. Increases between CA and CA in AD (0.32-fold, *P* = 6.04 × 10^−9^) and NT and *siPIP4K2A* (0.15-fold, *P* = 3. × 10^−4^) are noted. (**D**) qRT-PCR shows suppression of AR target gene *KLK3* in paired experiments where (**E**) intracellular pools of PI-4,5-P_2_ are detected with IF between LNCaP treated for 48 hours of 10 μM APA or DMSO control. Scale bars, 10 μm. (**F**) Image quantification of 50 cells per group showed elevated intensity of PI-4,5-P_2_ pool staining intensity in APA treatment. DAPI, 4′,6-diamidino-2-phenylindole. *t* test values: *P < 0.05; **P < 0.01; and ***P < 0.001.

Clues into how PCa transitions to CRPC may exist in understanding the metabolic adaptations of the prostate under conditions of AD. For example, the activity of cellular lysosome, the site of mTORC1 activation, increases under conditions of in vivo castration (*41*). In vitro, we found that a 48-hour treatment of APA prompts the accumulation of endolysosomes in LNCaP cells by 1.33-fold (Fig. 5, A and B). We question whether PI5P4Kα influences the metabolic adaptation of PCa to AD and used a high-coverage targeted lipidomics platform to characterize changes to key signaling lipids. LNCaP cultured in AD were compared to cells grown in complete serum conditions [RPMI 1640 + 5% fetal bovine serum (FBS)] for 48 hours. A total of 273 lipid species were semiquantified and assessed for changes based on lipid (sub)class and fatty acid chain composition (fig. S5, A and B). Figure S5C summarizes 53 lipids with levels that statistically differ between culture conditions. Six of seven assessed PI lipids were increased under AD conditions with an average of 141% higher relative fold lipid abundance (*t* test, *P* = 6.04 × 10^−9^; Fig. 5C). Androgen deprivation and AR inhibition has previously been shown to alter PI metabolism and membrane lipid concentrations (*42*, *43*). Next, we examined directly how down-regulating *PIP4K2A* affects the lipidome of PCa cells. The combined impact of siPIP4K2A in AD medium–enriched PI lipid species was higher than medium and NT controls alone (Fig. 5C). Paradoxically, inhibition of *PIP4K2* isoforms is reported to increase the total cell content of the PI-4,5-P_2_ lipid, but this increase is suggested to be in cell membrane–localized pools (*44*). Disruption to metabolic function can markedly shift membrane lipid pools leading to the question of how PI dysregulation could expose survival vulnerabilities in cancer cells. Notably, PI-4,5-P_2_, a species of phosphorylated PI, is an essential substrate activating some compensatory pathways that allow the survival through the progression to CRPC (*45*, *46*). With known importance, this raised the question of whether PCa depends on specific pools of PI to cope with cell stress.

### Intracellular PI-4,5-P_2_ is enriched following AR pathway inhibition

Recently, we have shown that the PI-4,5-P_2_ produced by PI5P4K is important for regulating cell metabolism (*9*). To query whether PI-4,5-P_2_ is modified under conditions of AR down-regulation, we used immunofluorescent (IF) staining protocols. Most of the PI-4,5-P_2_ are found on the cell surface membrane, and it is a critical lipid substrate for the PI3K/AKT (type 1 PIP kinase) pathway (*47*). However, alterations to type 1 alone do not account for the entire interplay of PI in intracellular metabolic compartments. A method to exclusively detect intracellular pools of PI-4,5-P_2_ using IF staining has previously been established (*9*, *48*). LNCaP cells treated with APA for 48 hours show strong suppression of AR target gene *KLK3* (Fig. 5D). While the AR pathway is suppressed, pools of intracellular PI-4,5-P_2_ were found to be enriched using IF intensity (Fig. 5, E and F). PI-4,5-P_2_ enrichment following APA treatment also increased the number of foci per cell (fig. S5D). Intracellular PI-4,5-P_2_ is a key signaling molecule for pathways involved with metabolic stress, and its enrichment may be functionally important for PCa disease progression. PCa relies on the AR pathway for growth; therefore, stress compensatory pathways involving intracellular PI-4,5-P_2_ could support cell survival during AD.

### PI5P4Kα localizes to the endolysosome and influences gland volume following in vivo androgen depletion

**Fig. 6.**
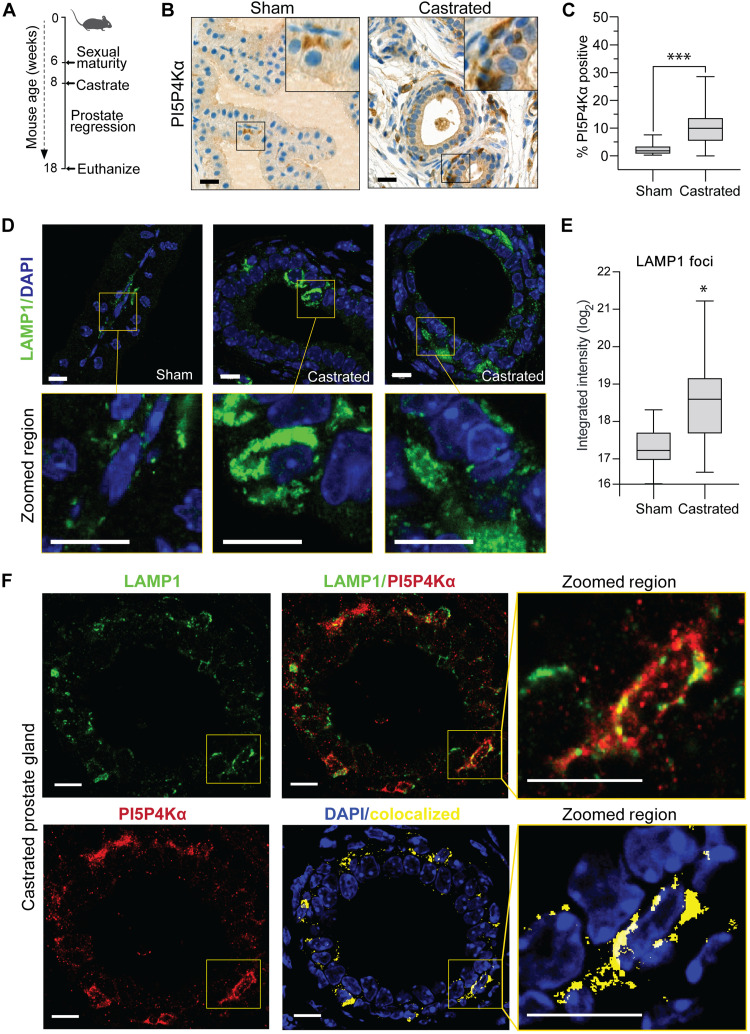
Lysosome localized PI5P4Kα is linked to castration adaptation of prostate tissue. (**A**) C57BL/6J mice were aged to 8 weeks, surgically castrated or used for control sham surgery procedure. Tissues were harvested 10 weeks after surgery. (**B**) PI5P4Kα detection was optimized and characterized in normal adult mouse prostate. Examples of positive 3,3′-diaminobenzidine (DAB) staining by immunohistochemistry are shown (scale bars, 20 μm) and (**C**) quantified between sham and castrated tissue groups as a percentage of positive DAB cells per number of cells counted per gland (*P* = 5.18 × 10^−9^). (**D**) Representative images of normal mouse prostate compared to castrated tissue when stained with LAMP1 endolysosome marker with IF-IHC. Scale bars, 10 μm. (**E**) Quantification of Lamp1 detection in castrated versus sham surgery mouse prostate tissue (Welch’s *t* test, *P* = 0.0193). DAPI nuclear stains are used as positive cell detection marks for IF assays. (**F**) IF staining of normal adult mouse prostate for colocalization of LAMP1 (green) and PI5P4Kα (red). Yellow indicates areas of colocalization. Scale bars, 10 μm. See fig. S7 for Zeiss software colocalization statistics. DAPI nuclear stains are used as positive cell detection marks for IF assays. *t* test values: *P < 0.05; and ***P < 0.001.

Targeting PI5P4Kα expression influences AR signaling in vitro, thereby prompting us to explore this relationship in vivo. We queried how AD resulting from surgical castration affects PI5P4Kα expression in mouse prostate. Eight-week-old C57BL/6J mice were castrated or subjected to a control sham procedure then harvested 10 weeks following surgery (Fig. 6A). PI5P4Kα protein expression was first confirmed in all lobes of the mouse prostate by Western blot (fig. S6A), and then, IHC and *Pip4k2a* RNA-ISH was used to positively detect PI5P4Kα patterns in tissue (fig. S6B). The mass of harvested urogenital (UG) tissue was markedly reduced in castrated animals as expected (fig. S6C); in addition, loss of nuclear AR staining in the prostate luminal epithelium was observed (fig. S6D). Whole castrated glands and an equal number of representative sham prostate regions were quantified for the number of high PI5P4Kα-positive stained cells. This pattern of PI5P4Kα staining is primarily observed along the luminal cell basal membrane and in the basal cell compartment (Fig. 6B). Sham tissue had 2.3% of prostate cells expressing high PI5P4Kα staining, while 11.13% of cells were positive in castrated glands (Fig. 6C). A relative enrichment of PI5P4Kα occurs in castrated mouse prostate.

Targeted suppression of *PIP4K2* results in an increase of lysosome cell content potentially through dysregulation of lysosomal membrane contacts with other organelles (*10*, *44*). In addition, lysosomes are involved in survival adaptation programs such as autophagy and mobilization of nutrients in the absence of AR pathway support (*49*). To determine whether lysosome regulation is altered in our model of AD, prostate tissue sections were stained for Lysosomal Associated Membrane Protein 1 (LAMP1). The cytoplasmic volume of prostate cells is atrophic compared to sham controls; however, the IF signal intensity of LAMP1 foci was elevated in the atrophic luminal cells of castrate surgery prostate glands (3.2-fold increase; Welch’s *t* test, *P =* 0.019; Fig. 6, D and E). Furthermore, we assessed whether PI5P4Kα protein expression correlates with patterns of LAMP1 positivity. LAMP1 positivity is in direct intracellular proximity with PI5P4Kα protein and has some areas of colocalization detected from IF confocal image analysis (Fig. 6F and fig. S7, A and B). Antibodies testing markers of the Golgi apparatus and ER were also tested but not found to correlate with focal PI5P4Kα expression patterns. Transmission electron microscopy images confirmed that there are concentrated lysosome numbers in mouse basal cells (fig. S7C). These data tie together PI5P4Kα with the regulation of the endolysosome and suggests a potential relationship with mTORC1, a complex highly dependent on lysosomal dynamics.

Under conditions of castration, lysosome-meditated degradation is used to catabolize cellular material of luminal prostate cells. Next, we questioned whether PI5P4Kα affects prostate tissue preservation under androgen deprivation conditions. A prostate-specific genetically engineered mouse (GEM) model was generated using a probasin (PB)–driven Cre recombinase combined with a homozygous *Pip4k2a* floxed (fl) allele (*Pip4k2a^fl/fl^*). PB-*Pip4k2a^fl/fl^* indicates allele deletion in the context of luminal prostate cell PB expression. No malignancy was observed in prostate tissues aged up to 300 and 400 days with either heterozygote or homozygote deletion of *Pip4k2a* (fig. S8, A to D). The only histomorphological features seen in a notable number of cases included (i) rare mitosis, (ii) rare individual cell apoptosis, and (iii) multifocal vacuolar cytoplasmic changes of prostate luminal epithelial cells. None of these features appeared to have been associated with a specific condition. An observed prostate luminal epithelial cytoplasmic vacuolation is compatible with a degenerative feature known to occur as an age-related change in laboratory mice.

**Fig. 7.**
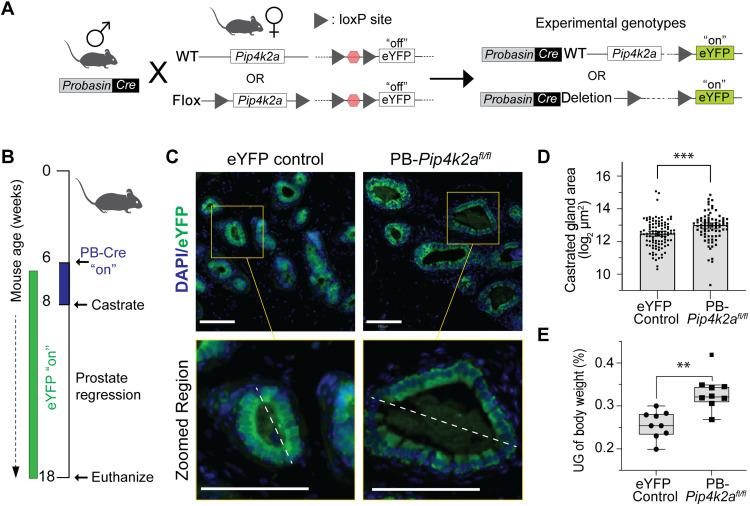
PI5P4Kα affects prostate gland regression during androgen deprivation. (**A**) Prostate-specific GEM model with R26eYFP reporter gene. PB-driven Cre recombinase system is used to delete *Pip4k2a^fl/fl^* alleles at time of mouse sexual maturity. An eYFP fluorophore is permanently “turned on” from Cre recombinase activation. WT, wild type. (**B**) Graphical summary of in vivo androgen deprivation experiment using mouse castration. *PB-Cre^+^;R26eYFP* control and *PB-Cre^+^;R26eYFP;Pip4k2a^fl/fl^* mice were aged to sexual maturity to activate Cre recombinase system, which can be detected with eYFP Cre reporter. Male mice were castrated at 8 weeks of age and harvested 10 weeks following surgery. (**C**) Representative images of GFP/eYFP detection with IF-IHC staining of castrated tissues. PB-expressing luminal cells show diffuse cytoplasmic GFP/eYFP staining of glandular epithelium. Scale bars, 100 μm. (**D**) Quantification of gland area of eYFP^+^ glands following surgical castration. Data are represented as median with Mann-Whitney test for significance (*P* = 0.0002). DAPI nuclear stains are used as positive-cell detection marks for IF assays. (**E**) Castrated UG mass relative to total mouse body weight shows an average of 0.26% for eYFP controls (*PB-Cre^+^;R26eYFP*; *n* = 9) compared to 0.32% for *Pip4k2a^fl/fl^* animals (*PB-Cre^+^;R26eYFP Pip4k2a^fl/fl^*; *n* = 8, one outlier, *t* test, *P* = 0.0004). *t* test values: **P < 0.01; and ***P < 0.001.

Including an R26eYFP Cre reporter allowed lineage tracking of mutant prostate cells following Cre activation from endogenous hormone signaling at the onset of sexual maturity (6 to 8 weeks; Fig. 7, A and B). IF staining of castrated tissue showed expression of enhanced yellow fluorescent protein (eYFP) Cre reporter in luminal prostate cells from both eYFP-only control and PB-*Pip4k2a^fl/fl^* genotypes. eYFP-expressing glands from PB-*Pip4k2a^fl/fl^* prostates had a greater remaining gland epithelium area, which could explain the elevated UG mass of these tissues (Fig. 7, C and D). On average, castrated PB-*Pip4k2a^fl/fl^* prostates had higher UG mass with a percentage relative to body weight of 0.32% compared to 0.26% of eYFP-only control animals (*t* test, *P* = 4.0 × 10^−4^; Fig. 7E). Together, these data could imply that inhibiting PI5P4Kα disrupts prostate tissue degradation under in vivo androgen deprivation conditions.

### Depletion of PI5P4Kα exposes survival vulnerability in models of PCa

**Fig. 8.**
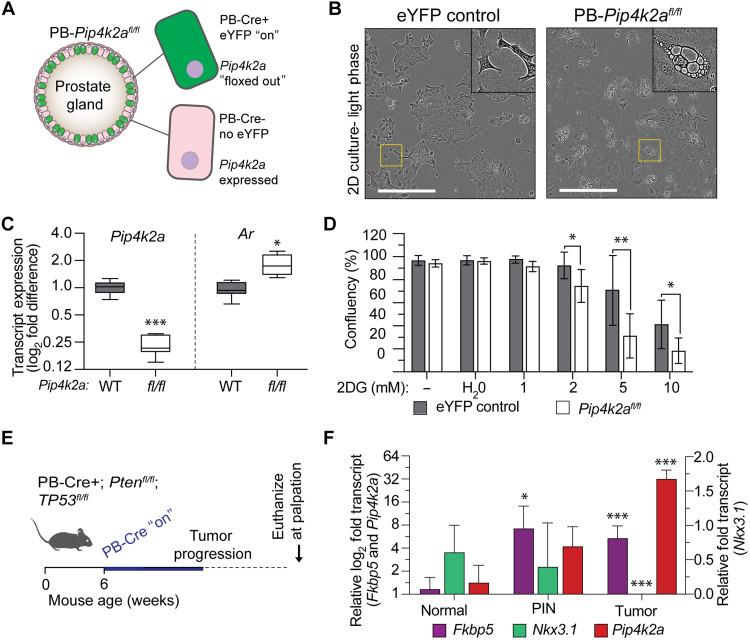
*Pip4k2a* is elevated in GEM cancer models, and deletion reduces prostate cell metabolic stress survival. (**A**) Graphical representation of *PB-Cre^+^;R26eYFP;Pip4k2a*^fl/fl^ prostate luminal cells with and without Cre recombinase activation and eYFP reporter expression in PB-expressing cells. (**B**) Light phase microscopy image of *PB-Pip4k2a*^fl/fl^ organoids cells in 2D culture demonstrates vacuole morphologic features. Relative to eYFP^+^ control cells. Scale bars, 400 μm. (**C**) qRT-PCR gene expression shows that *Pip4k2a* ^fl/fl^ cells have less *Pip4k2a* transcript (0.23-fold, *P* = 1.32 ×10^−6^) and have an increase in *Ar* (1.83-fold, *P* = 0.002). (**D**) *PB-Pip4k2a*^fl/fl^ cells were treated with 1, 2, 5, or 10 μM of 2DG or H_2_O control for 72 hours. Differences in cell confluency are represented compared to eYFP^+^ only control cells from three independent experiments. (**E** to **F**) Transcript expression was measured from mouse anterior prostate from animals with normal prostates, genotypes, and histology indicating early progression of prostatic intraepithelial neoplasia (PIN) and tumors from *PB-Cre^+^; Pten^fl/fl^; Trp53*^*fl*/fl^ prostates. Ar signature genes, *Nkx3*.1 (0.01 tumor, *P* = 2.55 ×10^−8^) and *Fkbp5* (7.2-fold in PIN, *P* = 0.01; 5.4-fold in tumor, *P* = 6.19 × 10^−5^) increase with early tumor development but are lost in advanced-stage disease. *Pip4k2a* is elevated in prostate tumors (32-fold in tumor, *P* = 2.52 ×10^−7^). *t* test values: *P < 0.05; **P < 0.01; and ***P < 0.001.

PI5P4Ks are promising targets for cancer therapeutic development in recent preclinical reports (*8*). Therefore, we first questioned whether vulnerabilities result from genetic *Pip4k2a* deletion. Noncancer organoids were isolated from mouse prostate tissue using the cell sorting for the R26eYFP reporter (Fig. 8A). Compared to wild-type controls, cell cultures generated from PB*-Pip4k2a^fl/fl^* prostate tissue had distinct vacuolized morphology in two-dimensional (2D) organoid culture (Fig. 8B), a phenotype associated with metabolic stress, lipid accumulation, and lysosome dysregulation (*50*, *51*). Transcript expression confirmed down-regulation of *Pip4k2a* with a subsequent increase in AR expression (Fig. 8C). Isolation of cells from PB*-Pip4k2a^fl/fl^* mouse prostates did not demonstrate major differences in cell proliferation compared to controls (*Pip4k2a^wt^*); however, when metabolically challenged with a glycolysis inhibitor, 2-deoxy-d-glucose (2DG), organoid cells had a greater reduction in proliferation (Fig. 8D). The role of *Pip4k2a* was further explored in a mouse model of PCa. *Pip4k2a* was up-regulated in aggressive *PB-Cre^+^;Trp53^fl/fl^;Pten^fl/fl^* prostate tumors and was inversely correlated with the AR gene expression signature (Fig. 8, E and F).

**Fig. 9.**
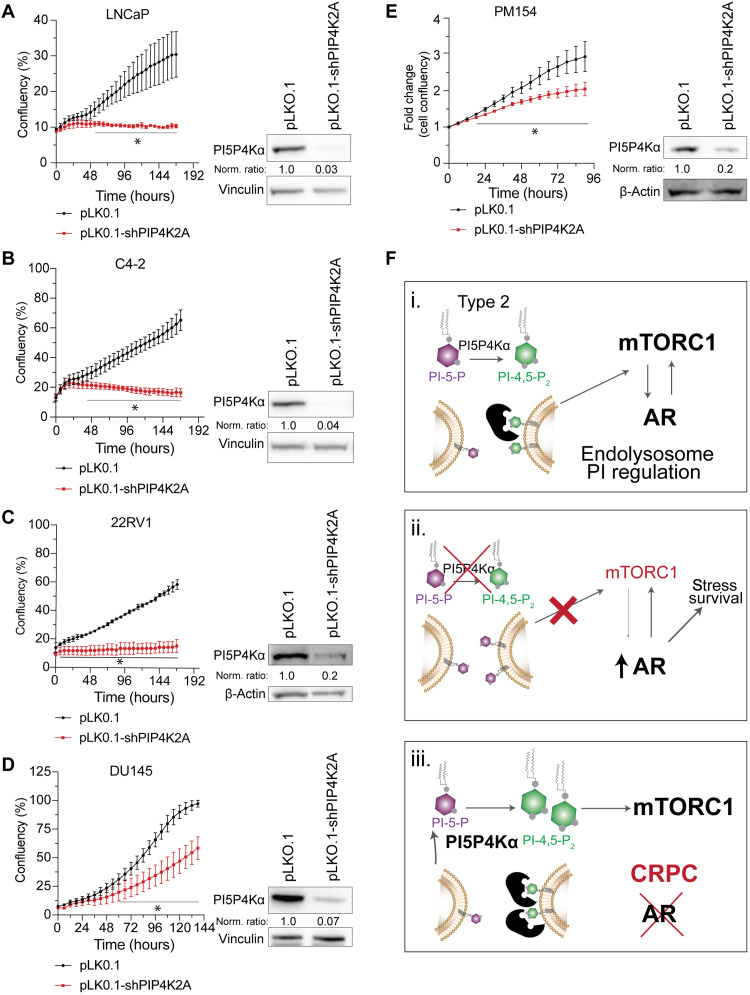
Acute depletion of *PIP4K2A* exposes survival vulnerability in models of PCa. Human PCa cell lines are impaired in proliferation when infected with stable *PIP4K2A-*targeted shRNA (shPIP4K2A) construct relative to control pLKO.1 lentivirus. PI5P4Kα knockdown confirmed by Western blots and cell proliferation changes measured by changes in confluence over time shown for (**A**) LNCaP, (**B**) C4-2, (**C**) 22RV1, and (**D**) DU145, which represents a model of castrate-resistant PCa (CRPC). (**E**) Changes in fold growth over time shown for the 3D Neuroendocrine PCa (NEPC) organoid line PM154. *t* test values: *P < 0.05 . Data are represented as means ± SD. (**F**) Graphical representation of the proposed mechanism. PI5P4Kα is an important regulator of metabolic organelles from the endolysosome system, which regulates the nutrient sensing and metabolic stress pathway driven by mTORC1. In PCa, AR and mTORC1 are established to have a dynamic interplay that is critical in prostate cell growth and survival. (i and ii) We show that the depletion of *PIP4K2* increases PCa cell stress and results in the up-regulation of AR and suggest that this is mitigated via the down-regulation of mTORC1. (iii) Patient data suggests an increase in *PIP4K2A* in AR-independent PCa cases (CRPC). PI5P4Kα may become critical in maintaining CRPC growth by promoting mTORC1 and thereby exposes a vulnerability in the cancer metabolism.

Next, we asked whether acute PI5P4Kα depletion affects the proliferation of human PCa models representative of different stages of disease. Lentiviral-mediated shRNA depletion of *PIP4K2A* enhances and sustains a knockdown beyond transient siRNA tools. A pLKO.1-GFP-shRNA lentiviral construct paired with green fluorescent protein (GFP) selection demonstrates that sustained *PIP4K2A* depletion prevents the viable proliferation of LNCaP, C4-2, and 22RV1 cells while reducing the doubling time of DU145 (control, 44.93 hours; shPIP4K2A, 48.80 hours; Fig. 9, A to D). In addition to cell lines, the PM154 organoid model of advanced PCa also demonstrated strong growth reduction following lentiviral delivery of shPIP4K2A (doubling time control, 62.45 hours; shPIP4K2A, 95.56 hours; Fig. 9E)

Lastly, down-regulation of *PIP4K2A* in LNCaP cells enriched the proportion of G_1_ cycle–arrested cells (fig. S9, A and B). *PIP4K2A^Cas9mut^* LNCaP cells demonstrate reduced proliferative ability following low-dose treatment of 0.5 μM BKM120 (fig. S9C), as well as when exposed to 5 and 10 μM APA (fig. S9D). Together, targeting *PIP4K2A* exposes a vulnerability in PCa through the dysregulation of key metabolic pathways.

## DISCUSSION

The treatment of advanced PCa requires more than a mutational profile; clinicians need actionable interventions founded on functional prostate studies. We propose targeting a previously unexplored lipid kinase for PCa, PI5P4Kα. Here, we are the first to question how PI5P4K is expressed and functions in prostate luminal biology. We show that (i) *PIP4K2A* expression is enhanced in PCa with AR signaling reflecting CRPC, (ii) PI5P4Kα localizes to the lysosome where its influence on mTORC1 signaling may affect AR, and (iii) inhibiting PI5P4Kα exposes PCa cells to a metabolic vulnerability that may enhance efficacy of targeted therapeutics. This line of evidence prompts a model where, under AR high conditions, PI5P4Kα supports basal mTORC1 signaling and becomes more important as PCa cells metabolically adapt to androgen independence. CRPC relies on up-regulating mTORC1 for growth and survival through therapeutic stress; therefore, it is beneficial to increase the expression of enzymes like PI5P4Kα to orchestrate pathway dynamics (Fig. 9F).

We sought to examine whether PI5P4Kα inhibition affected AR and found an inverse relationship that we propose is mitigated via mTORC1. Previous literature strongly supports the involvement of the PI5P4K isoforms in promoting metabolic stress survival through metazoan mTORC pathways. A single *PIP4K2* ortholog exists in the model organisms *Caenorhabditis elegans*, *Drosophila*, and zebrafish (*Danio rerio*), whereas mice and humans share three isoforms (α, β, and γ) (*2*, *3*, *10*, *52*, *53*). Gupta *et al*. (*52*) demonstrate that *PIP4K2* mutant *Drosophila* have reduced mTORC1 signaling and growth that can be rescued with overexpression of the mTORC1 activator, Rheb. *C. elegans* lacking PPK-2 have a decreased life span and impaired lipid droplet recycling, a phenotype linked to dysfunctional lysosomal autophagy (*10*). Similarly, in mouse models, genetic targeting of *Pip4k2a/b* also altered lysosome-autophagosome fusion, resulting in mTORC1 suppression, liver lipid droplet accumulation, increased lysosome content, and defeats in lipid metabolism (*9*, *10*). Using human cells, targeting PI5P4Kα/β inhibits mTORC1 phospho-target expression and exposes metabolic vulnerability to sarcoma and breast cancer cell survival under conditions of nutrient stress (*9*, *54*). These observations are not restricted to the α and β isoforms, as PI5P4Kγ is reported to maintain a regulatory loop with mTORC1 to control activation in conditions of low nutrients (*55*). While less is known about the role of PI5P4K specifically in cancer biology, one hypothesis is that transformed cells could hijack PI5P4K to reprogram mTORC1 to maintain activation of cancer growth. In PCa, this may be particularly advantageous as AR-sensitive adenocarcinoma adapts to a “fuel switch,” as it lacks lipogenesis pathway stimulation following AD.

In this study, we associate elevated *PIP4K2A* expression with AR-independent PCa (i.e., CRPC). Targeting PI5P4K could offer a strategy to control mTORC1, as advanced PCa is highly reliant on the activation of mTORC1 for growth and survival. For example, the gene for the leucine transporter, LAT3, was first characterized because of its high overexpression in PCa (*56*). Leucine is an essential activator of the mTORC1 complex, and PCa up-regulates amino acid transporters with the purpose of meeting demands for supporting tumor growth (*57*). Furthermore, a reciprocal relationship has been previously established between AR and mTOR activity. Multiple studies report sub-baseline mTOR activation from nutrient stress or rapamycin-increased AR protein levels (*36*, *58*). The precise mechanism coordinating this feedback is up for debate and could involve transcriptional regulation of mTOR repressors TSC Complex Subunit 1 and 2 (TSC1/2) by AR (*58*), deactivation of the tumor suppressor phosphatase PH Domain And Leucine Rich Repeat Protein Phosphatase 1 (PHLPP1) (*59*), reactivation of AKT via mTORC2 (*36*), or reactivation of insulin receptor substrate 1. Therefore, it is expected that most mTOR inhibitors as monotherapies have not succeeded past phase 2 clinical trials in PCa. This is thought to be due to a high degree of reciprocal feedback loops involving PI3K and AR that may function to develop drug resistance (*23*). It is presently accepted that therapeutic strategies that involve the inhibition of multiple arms of the PI3K-mTOR-AR network may offer the greatest promise in minimizing the risk of recurrence.

To our knowledge, this study is the first to directly show the colocalization of PI5P4Kα at the lysosome in tissue. The role of PI5P4K is especially context dependent; specifically, it varies between different cell types and the status of nutrient availability. The added complexity is layered onto the mechanistic details of the PI5P4K mode of action if also considering kinase-dependent or independent functions (*44*). One limitation of this study is that we do not directly address whether scaffolding functions of PI5P4K are involved in its relationship with AR signaling. While not presently in the scope of our investigation, further work comparing kinase inhibitors versus PI5P4K protein degraders will be valuable in demonstrating exact functions that PI5P4K relies on to support cancer metabolism.

mTORC1 is considered the “master regulator” of cell metabolism, and it is necessary for this protein complex to be localized to the surface membranes of the intracellular organelles, the lysosome, and endosome (*60*, *61*). Endomembrane PIP lipids have major implications in mTORC1 regulation. Class II PI3K ß (PI3KC2ß) synthesizes PI-3,4-P_2_ at the late endosome, which can repress mTORC1 activity through recruitment of inhibitory binding proteins to Raptor (*62*). Recently, PI-3,5-P_2_ and PI-4,5-P_2_ were shown to be required for mTORC1 regulation at the lysosome surface through TSC translocation and complex binding (*16*). Although a very minor fraction of phospholipids, the PIPs embedded into lipid membranes within and surrounding the cell regulate a large number of molecules that have a variety of functions [reviewed in (*63*)]. There is still limited knowledge regarding the full spectrum of signaling cascades that are orchestrated by PIP lipids in PCa. Goto *et al*. (*64*) nominate PIP lipids as potential PCa biomarkers after conducting the first spatial high-resolution matrix-assisted laser desorption/ionization imaging mass spectrometry analysis of benign versus cancer patient tissue. Recently, shifts in PIP abundance and acyl chain saturation have also been associated with PTEN mutant PCa (*45*). A recurring limitation to most published studies involving PIPs in cancer, however, is the technical challenge in deciphering individual PI classes (PI, PIP_1_, PIP_2_, and PIP_3_) and regioisomers (PI-4,5-P_2_ versus PI-3,4-P_2_).

More than 150 kinase-targeted drugs (mostly protein kinases) are currently in cancer clinical trials, with about 37 having received U.S. Food and Drug Administration approval since the 1980s (*65*). In May 2019, alpelisib, a PI3K inhibitor, was the first of its kind to be approved for breast cancer. Kinases are key signaling molecules that offer effective drug targets in a pharmaceutical environment already adapted for their development. Interest in the PI5P4Ks is growing, as seen with the publication of a pan isoform covalent inhibitor (THZ-P1-2) (*40*), a dual PI5P4Kα/β inhibitor (*54*) and earlier single-isoform targeted compounds (*66*, *67*).

In conclusion, this is the first report to link PI5P4Kα to the AR pathway and its regulation in PCa. Evidence reported here opens the question of whether PI5P4Kα is vital for maintaining mTORC1 activation under stress conditions, specifically at the endolysosome, which relies heavily on PI to mitigate organelle dynamics. We propose that the influence of PI5P4Kα on lysosomal mTORC1 is responsible for stress activation of AR and exposes survival vulnerability in cancer. Herein prompts the rationale for the therapeutic development of PI5P4K-targeted inhibitors for the multimodal treatment of PCa.

## MATERIALS AND METHODS

### AR signaling score and mTOR score analysis of patient datasets

AR signaling score was calculated as previously described, namely, by calculating the Pearson’s correlation coefficient between the log_2_-transformed fragments per kilobase of exon per million mapped fragments (FPKM) values of a set of 30 AR-regulated genes and a reference vector (tables S1 and S2) (*68*). The mTOR signaling score was calculated as previously described (*17*). Briefly, a *t* test was performed between the log_2_-transformed FPKM values of genes up-regulated on mTOR pathway activation, against those down-regulated on mTOR pathway activation. The absolute value of the resulting *t* statistic was then used as the “mTOR score” for each sample.

### Cell models and culture conditions

LNCaP [male; American Type Culture Collection (ATCC), RRID: CVCL_1379], C4-2 (male; ATCC, RRID: CRL-3314), VCaP (male; ATCC, RRID: CRL-2876), PC3 (male; ATCC, RRID: CRL-3470), DU145 (male; ATCC, RRID: CVCL_0105), 22RV1, and RWPE (male; ATCC, RRID: CVCL_3791) cells were acquired from the ATCC. LNCaP-AR cells were a gift from Charles L. Sawyers and Ping Mu (Memorial Sloan Kettering Cancer Center) (*34*). LNCaP were grown in RPMI 1640 (Gibco, A1049101) supplemented with 5% FBS (Gibco, 10270106) and 1% penicillin-streptomycin (Gibco, 11548876) on poly-l-lysine–coated plates. VCaP, PC3, DU145, and 22RV1 were maintained in Dulbecco’s modified Eagle’s medium (Gibco, 31966021), supplemented with 10% FBS and 1% penicillin-streptomycin. RWPE were maintained in keratinocyte serum-free medium (Gibco, 17005075) supplemented with bovine pituitary extract and human recombinant EGF (included) and 1% penicillin-streptomycin (Gibco, 11548876). Androgen deprivation (AD) was modeled in vitro by supplementing RPMI 1640, lacking phenol red (Fisher Scientific, 11-835-055) with 5% C/S serum (Thermo Scientific, A3382101) and penicillin-streptomycin. All cell lines were grown at 37°C with 5% CO_2_, regularly tested for mycoplasma and authenticated using short tandem repeat (STR) profiling.

### GSEA and RNA-seq

Cell line transcript data were acquired from the Cancer Cell Line Encyclopedia (*69*) database, and organoid cell models were sequenced as reported (table S3) (*33*, *70*). Bulk RNA-seq was conducted on RNA extracted from phosphate-buffered saline (PBS)–washed cell pellets using a QIAGEN RNeasy kit. RNA was deoxyribonuclease-treated (Ambion, AM1906) and quality-assessed using Bioanalyzer and Qubit. Sequencing libraries were prepared using 500 ng of RNA and Illumina TruSeq Stranded mRNA with IDT for Illumina TruSeq UD RNA indexes. FASTQ read files were analyzed as previously described (*70*) using STARv2.3.0e (*71*), RSEQtools (*72*), and GENCODE v19 (www.gencodegenes.org/human/release_19.html). GSEA analysis was conducted as previously described (*9*).

### Chemical inhibitors and cell proliferation assays

A summary of key reagent product information is listed in table S4.

#### siRNA targeted knockdown

Cell lines were plated on poly-l-lysine–coated six-well plates and then transfected with pooled siRNA targeting *PIP4K2A* (siPIP4K2A) or NT control oligos (ON-TARGETplus, Dharmacon) using recommended conditions of the Lipofectamine RNAiMAX Transfection Reagent (Thermo Fisher Scientific, 13778150). The next day, we switched to C/S medium and light RPMI 1640 + 5% C/S FBS for 48 hours for AD conditions.

#### Inhibitor treatment

Cells were treated with 5 or 10 μM of APA (Selleck Chemicals, S2840), 1 nM rapamycin (Selleck Chemicals, S1039), or 10 μM BKM120 (Selleck Chemicals, S2247) with dimethyl sulfoxide (DMSO) solvent control in culture medium in either six-well or 96-well plate formats. Inhibitor(s) efficacy was confirmed with quantitative real-time polymerase chain reaction (qRT-PCR) or Western blot of known downstream targets. Glycolysis inhibition was done using 2DG (Sigma-Aldrich, D8375) in sterile H_2_O at indicated dilutions. DHT (Sigma-Aldrich, 10300) treatments were done for 18 hours in AD medium, which is made up of 5% C/S serum. Ethanol was used for DHT solvent control.

#### Cell proliferation

Cell models were seeded on poly-l-lysine–coated 96-well plates at 5000 cells per well between three and six replicates per condition per experiment repeat. Cell confluence was determined using the IncuCyte S3 instrument and the IncuCyte S3 2018B software (Essen Bioscience, Germany). Changes in proliferation over time are estimated on the basis of the digital area mask coverage of field of view from microscope images captured at ×20 magnification.

### Lentiviral hairpin systems

Plasmids used were previously generated for stable shRNA knockdown of *PIP4K2A* (pLKO.1-GFP-shPIP4K2A and control pLKO.1-GFP) (*10*) and doxycycline-induced knockdown of *PIP4K2A* (Ez-tet-Puro-Scramble, Ind5145, and Ind6009) (*9*). Lentivirus was produced as previously reported using transfection of human embryonic kidney 293T cells. The target cell population was infected with 1:4 volume virus:medium ratio in medium containing polybrene (0.8 μg/ml), then centrifuged at 1000*g* for 30 min, and left to recover for 48 hours in a cell culture incubator. Selection was conducted using fluorescence-activated cell sorting (FACS) for GFP^+^ cells.

### Western blot protein detection

Protein lysates were extracted from PBS-washed cell pellets using radioimmunoprecipitation assay with protease and phosphatase inhibitors. Total protein concentration was measured using the Pierce BCA Protein Assay Kit (Thermo Fisher Scientific). Between 20 and 50 μg of protein was used for standard SDS–polyacrylamide gel electrophoresis procedures with Mini-Protean TGX gels (4 to 15%; Bio-Rad, 456-1084). Membrane transfer to nitrocellulose was done using an iBlot2 quick transfer system (Thermo Fisher Scientific, IB23001). Blocking and secondary antibody incubations were done using 5% milk in Tris-buffered saline with 0.1% Tween^®^ 20 detergent (TBST), and primary antibodies were probed with optimized conditions in 5% BSA unless stated otherwise (table S5). A minimum of three 5-min wash steps in TBST (1% tween) were used between incubations and before visualization. Final detection with chemiluminescence was done with the Luminata Forte substrate (Thermo Fisher Scientific, WBLUF0100), and multiple image exposures were acquired with the FUSION FX7 EDGE Imaging System (Witec AG). Band densitometry was measured using ImageJ version 1.53a and normalized to housekeepers.

### qRT-PCR transcript detection

RNA was extracted from cell pellets using the QIAGEN RNeasy Kit (QIAGEN, 74106). cDNA was synthesized using the FIREScript RT cDNA Synthesis Kit (Solis BioDyne, 06-15-00200); then, qRT-PCR was conducted on a ViiA 7 system (Applied Biosystems) with the HOT FIREPol EvaGreen qPCR master mix (Solis BioDyne, 08-24-00020). Gene cycle thresholds were calculated using triplicate replicates and normalized to housekeeping controls. See table S6 for primer list and sequences.

### LysoTracker flow cytometry

LNCaP (3 × 10^5^) were treated with 10 μM of APA or DMSO for 48 hours. Cells were incubated with 50 nM LysoTracker Deep Red (Invitrogen), freshly prepared in RPMI 1640 + 5% FBS, for 1 hour in the dark at 37°C. At least 10 000 live cells were captured using ImageStream-X MKII flow cytometry and analyzed by the IDEAS version 6.2.

### Lipidomics analysis

#### Lipid extraction

Cell metabolism was quenched by the addition of cold 80% methanol (MeOH) following cell scraping. This methanolic solution of collected cells was evaporated, and lipids were extracted by the addition of butanol:MeOH (1:1) through mechanic homogenization (2 × 20 s in Precellys tissue homogenizer). Protein quantification was performed on the remaining cell pellets, and the total protein content was used for sample amount normalization.

#### Targeted analysis of lipids

Analysis on extract lysates was performed by hydrophilic interaction liquid chromatography coupled to tandem mass spectrometry (MS/MS) in both positive and negative ionization modes using a Q-TRAP 6500+ liquid chromatography–MS/MS (LC-MS/MS) system (Sciex Technologies) (*73*, *74*).

#### Quality control

Pooled quality control (QC) samples were analyzed periodically (every five to six samples) throughout the overall analytical run to assess the data quality, correct the signal intensity drift (if any), and remove the peaks with poor reproducibility [coefficient of variation (CV) > 30%]. In addition, a series of diluted QCs were prepared by dilution with butanol:MeOH: 100, 50, 25, 12.5, and 6.25% QC.

#### Data processing

Raw LC-MS/MS data were processed using the MultiQuant Software (version 3.0.3, Sciex Technologies). Relative quantification of metabolites was based on extracted ion chromatogram areas for the monitored multiple reaction monitoring (MRM) transitions. Signal intensity drift correction on peak area data was done within the LOWESS/Spline normalization program 8 (R software), followed by noise filtering [CV (QC features) > 30%] and visual inspection of linear response (*75*).

### Intracellular PI-4,5-P_2_ detection by IF

PI-4,5-P_2_ detection in intracellular membranes was done using IF protocols as previously described (*9*, *48*). Briefly, cells were plated and drug-treated on poly-l-lysine–coated coverslips in six-well dishes. At a final time point, cells were fixed in 2% paraformaldehyde (PFA) and then permeabilized with 20 mM digitonin. Samples were blocked in 5% normal goat serum in buffer A with 50 mM NH_4_Cl before being incubated in primary anti–PI-4,5-P_2_ (1:100; Abcam, ab11039) overnight. Samples were washed three times and then incubated with secondary antibodies, followed by postfixation with 2% PFA in PBS. DAPI (4′,6-diamidino-2-phenylindole) was added to the wash buffer during the final wash steps. Samples slides were imaged with 60× oil immersion on Zeiss LSM 710 confocal microscope. Images were quantified using the ImageJ Spot Localization ComDet plug-in. A minimum of 50 cells were quantified per condition per replicate.

### GEM models

All animal studies were approved by the Cantonal Veterinary Ethical Committee, Switzerland (license BE 41/2018). Animals were housed in ventilated cages with unrestricted access to presterilized food and fresh water. A maximum of five animals were maintained per cage on Aspen bedding. Ambient temperature was 20° ± 2°C, kept at a constant humidity of 50 ± 10%, and on a 12-hour automatic light-dark cycle. *Pip4k2a^tm1.2Lca^* animals (MGI:5568930), referred to as *Pip4k2a*^*fl/fl*^, were a gift of the group of L. Cantley. The generation of *Pip4k2a^fl/fl^* (*2*, *10*), *Probasin-Cre* (PB-Cre) (*76*), and *Rosa26eYFP* (*77*) mice has been previously described. These strains were then crossbred to generate the *PB-Cre^+/−^; R26eYFP^+/+^; Pip4k2a^fl/fl^* mice used in the mouse adult prostate and castration study. Breeding schemes limited inclusion of the PB-Cre^+^ allele exclusively through male mice to avoid off-target effects. Animals were genotyped using the Transnetyx real-time PCR platform.

### Castration surgery

Surgical castration or sham surgery was performed on 8-week-old C57BL/6JRj mice. Standard procedures are previously described (*78*). Mice are preemptively dosed with buprenorphine, and the surgical area was shaved following confirmation of suitable anesthetic plane using isoflurane and a 37°C heat pad. Marcaine (0.25 to 0.5%) solution was used for local anesthetic, and the surgical site was prepared with alternating 70% isopropyl alcohol and povidone-iodine scrub. A midline incision was made in the scrotum, and testicles were removed with cauterization. The incision site was closed using one sterilized wound clip (Autoclip), which was removed between 10 and 14 days postoperatively. Animals were monitored for the duration of the experiment, and analgesic treatments were applied for 3 days following the procedure. UG tissue mass, including prostate, bladder, and seminal vesicle was recorded, while prostate tissue was collected 10 weeks following castration or sham procedure.

### IHC detection

Tissue sections were processed using standard histology procedures, fixed with 4% formaldehyde overnight, and paraffin-embedded, and sections were cut at 2.5 μm. Antibody staining was optimized for PI5P4Kα (Proteintech, 1246-1-AP) with 30 min tris buffer antigen retrieval with 1:200 dilution antibody and with 30-min citrate buffer antigen retrieval. Conditions for additional antibodies are listed in table S5. Slide staining was done using the BOND RX autostainer (Leica Biosystems) as previously described (*75*, *79*)

### IF tissue staining

Adult C57BL/6JRj mouse prostates were harvested and paraffin-embedded. Tissue serial sections were deparaffinized in xylene and rehydrated using decreasing concentrations of ethanol (100 to 95 to 70% to H_2_O). Heat-induced epitope retrieval was used at a setting of 98°C for 35 min in tris-EDTA buffer. Slides were left to return to room temperature, then washed in Tris-buffered saline (TBS), blocked in 5% normal goat serum at room temperature, and probed with primary antibodies overnight at 4°C in an immunostain moisture chamber. Protein targets were detected using 1:200 PI5P4Kα polyclonal antibody (Proteintech, 1249-1-AP) and 1:200 LAMP1 monoclonal antibody (Abcam; Ab24871) with 1:300 goat anti-rabbit Cy3 (Jackson ImmunoResearch, 111-165-003) or donkey anti-rabbit A488 (Jackson ImmunoResearch, 712-545-150) secondary antibody. DAPI (100 ng/ml) was used as nuclear counterstain, and ProLong Gold (Thermo Fisher Scientific; P36930) was used for mounting. Images were acquired with 60× oil immersion on Zeiss LSM 710 confocal microscope.

### Digital tissue quantification

Whole-slide images were acquired on a Pannoramic 250 (3DHistech) slide scanner, and images were exported from CaseView software (version 2.5). File codes were used to blind the investigator from the experimental group during data acquisition and annotation. Sample group blinding was also implemented for pathology evaluation of tissue samples. Images were analyzed using QuPath v0.2 (*80*) and as previously described (*81*).

### Primary mouse organoid models

Mouse organoids were isolated as previously described (*82*). Adult prostates were harvested, dissociated overnight, and FACS-sorted for eYFP^+^ cell population from mouse lines containing activated *R26eYFP* Cre recombinase reporter. Organoids were maintained in 3D Matrigel culture and transferred to collagen-coated 96-well plates for 2D proliferation assays (as described above).

### Statistics and data availability

Data are expressed as means ± SD. Statistical analyses for all data, including microscopy quantification, qRT-PCR data, metabolomics, and lipidomics, were done using Student’s two-tailed *t* test or analysis of variance (ANOVA) as indicated using GraphPad Prism. Statistical significance is indicated in the figure legends [*P < 0.05; **P < 0.01; ***P < 0.001; and not significant (n.s.), *P* > 0.05] unless specified otherwise. The datasets generated during and/or analyzed during the current study are available from the corresponding author on reasonable request. RNA-seq, lipidomics, and Western blot source data are available in data S1 to S3.

## Notes

View/request a protocol for this paper from *Bio-protocol*.
