# Supplementary material for: PI5P4Kα supports prostate cancer metabolism and exposes a survival vulnerability during androgen receptor inhibition
Source: Sci Adv. 2023 Feb 1;9(5):eade8641. doi: 10.1126/sciadv.ade8641 (PMC9891700; doi:10.1126/sciadv.ade8641)
Supplement: Supplementary file 1 — Figs. S1 to S9 Tables S1 to S6 [file sciadv.ade8641_sm.pdf]

Science Advances
AAAS

Supplementary Materials for

PI5P4Kα supports prostate cancer metabolism and exposes a survival vulnerability during androgen receptor inhibition

Joanna Triscott *et al.*

Corresponding author: Mark A. Rubin, mark.rubin@unibe.ch;
Brooke M. Emerling, bemerling@sbpdiscovery.org

Sci. Adv. **9**, eade8641 (2023)
DOI: 10.1126/sciadv.ade8641

The PDF file includes:

Figs. S1 to S9
Tables S1 to S6
Legends for data S1 to S3

Other Supplementary Material for this manuscript includes the following:

Data S1 to S3

Figure S1.

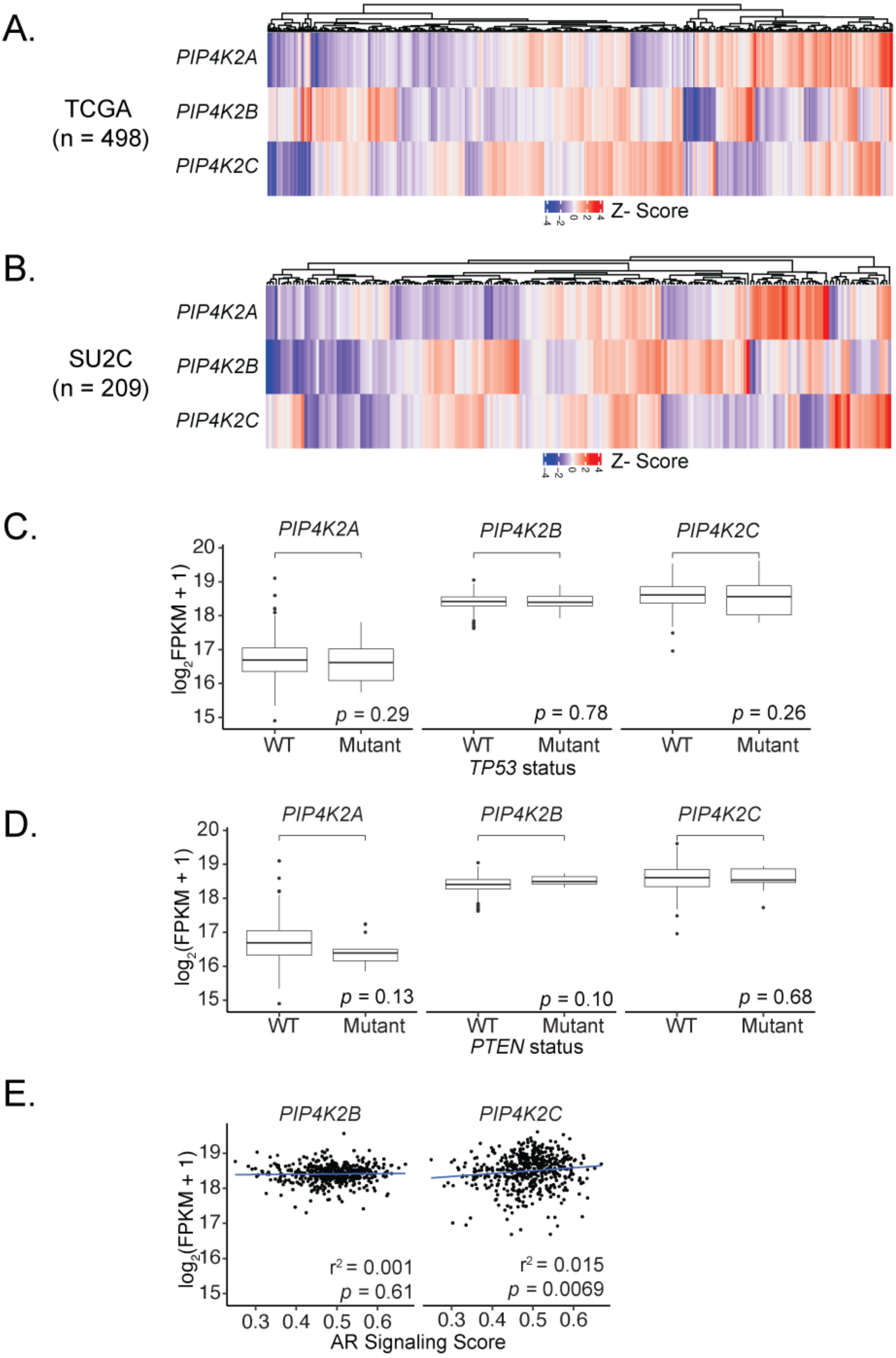

A.
TCGA
(n = 498)
PIP4K2A
PIP4K2B
PIP4K2C
Z- Score
B.
SU2C
(n = 209)
PIP4K2A
PIP4K2B
PIP4K2C
Z- Score
C.
PIP4K2A
PIP4K2B
PIP4K2C
log2(FPKM + 1)
p = 0.29
p = 0.78
p = 0.26
WT
Mutant
TP53 status
D.
PIP4K2A
PIP4K2B
PIP4K2C
log2(FPKM + 1)
p = 0.13
p = 0.10
p = 0.68
WT
Mutant
PTEN status
E.
PIP4K2B
PIP4K2C
log2(FPKM + 1)
r2 = 0.001
p = 0.61
r2 = 0.015
p = 0.0069
AR Signaling Score

Figure S1. Characteristics of *PIP4K2* in clinical patient databases. Analysis of gene expression of the *PIP4K2* isoforms was conducted on available transcript data from **(A)** The Cancer Genome Atlas (PRAD-TCGA) (N = 498) and **(B)** Stand Up to Cancer (SU2C) datasets (N = 209). No evidence of enrichment was found to correlate with the *PIP4K2* isoforms and **(C)** *TP53* or **(D)** *PTEN* mutation status. **(E)** No correlation is associated with *PIP4K2B* (r^2= 0.001, p = 0.61) or *PIP4K2C* (r^2= 0.015, p = 0.0069) and AR signaling score.

Figure S2.

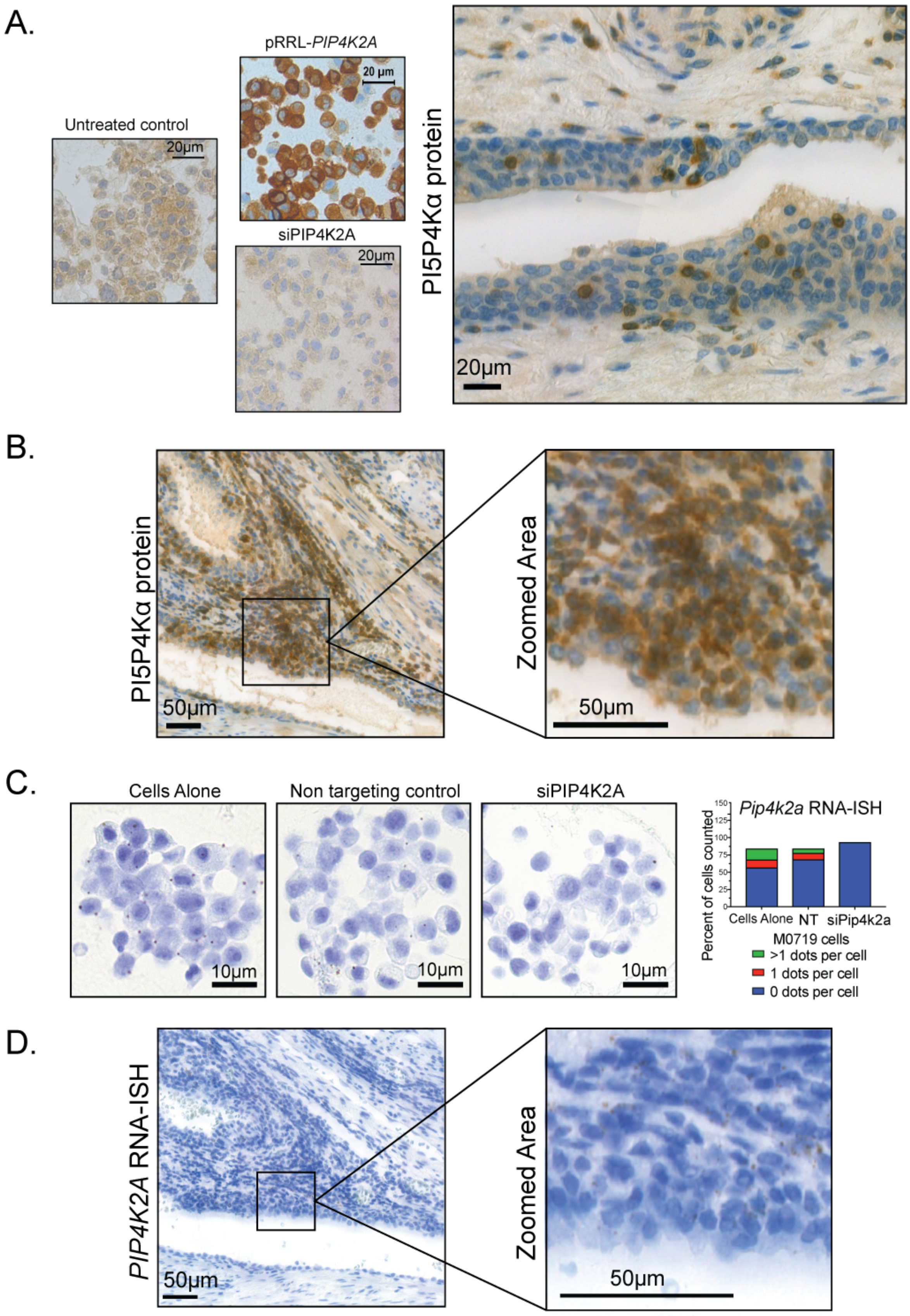

A.
Untreated control
20µm
pRRL-PIP4K2A
20 µm
siPIP4K2A
20µm
PI5P4Kα protein
20µm
B.
PI5P4Kα protein
50µm
Zoomed Area
50µm
C.
Cells Alone
10µm
Non targeting control
10µm
siPIP4K2A
10µm
Pip4k2a RNA-ISH
Percent of cells counted
150
125
100
75
50
25
0
Cells Alone
NT
siPip4k2a
M0719 cells
>1 dots per cell
1 dots per cell
0 dots per cell
D.
PIP4K2A RNA-ISH
50µm
Zoomed Area
50µm

Figure S2. PI5P4Kα expression in clinical samples. (A) Light phase images of immunohistochemical (IHC) antibody work up for PI5P4Kα. Human LNCaP cells either expressing pRRL-*PIP4K2A* overexpression construct or knocked down for *PIP4K2A* using siRNA (siPIP4K2A) were embedded in paraffin, section and stained with PI5P4Kα (ProteinTech, 12469-1-AP). IHC staining of human PCa patient tissue demonstrates positive detection of PI5P4Kα protein in regions of basal cell hyperplasia. **(B)** IHC staining of human PCa patient tissue demonstrates positive detection of PI5P4Kα in leukocytes in regions of tumor inflammation. **(C)** Validation of RNA-in situ hybridization (ISH) probe for *Pip4k2a* using mouse organoid cells. Cells that were cultured in 2D then treated with siPip4k2a or control non-targeting. Then pelleted cells were embedded in paraffin, sectioned, and probed with RNA-ISH. Image quantification measured number of positive ISH spots per cell per condition. As well, **(D)** RNA-ISH staining of human PCa patient tissue demonstrates positive detection of *PIP4K2A* in regions of tumor inflammation.

Figure S3.

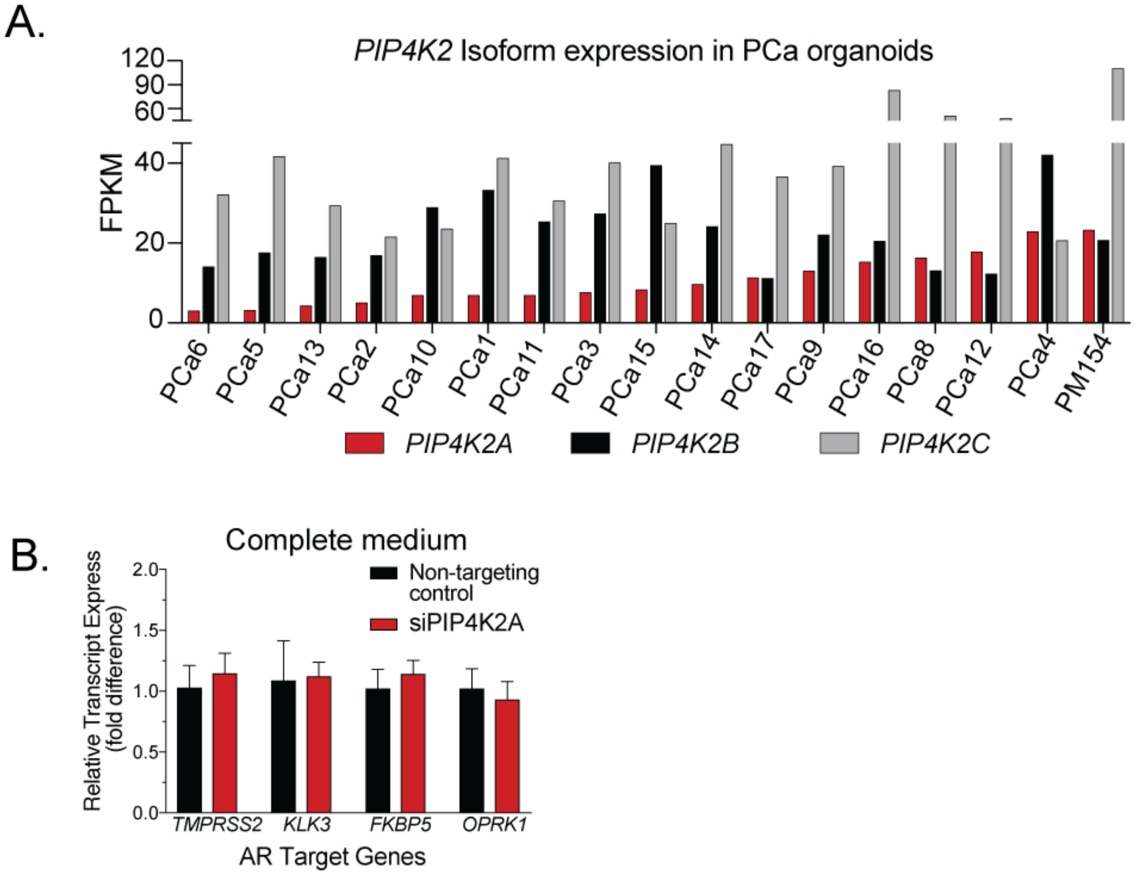

Figure S3. *PIP4K2* is expressed in PCa and knock down impacts AR signaling in AD. (A) Transcript expression of *PIP4K2A, PIP4K2B, and PIP4K2C* shown as fragments per kilobase of exon per million mapped fragments (FPKM) from RNA sequencing of PCa organoid lines. **(B)** Gene expression of AR target genes (*TMPRSS2, KLK3, FKBP5*, and *OPRK1*) were measured with qRT-PCR in LNCaP cells transfected with siRNA targeting *PIP4K2A* or non-targeting (NT) control (72hr) in complete RPMI medium (+5% FBS). *t* test values: n.s., not significant ($p > 0.05$), * $p < 0.05$, ** $p < 0.01$, *** $p < 0.001$.

Figure S4.

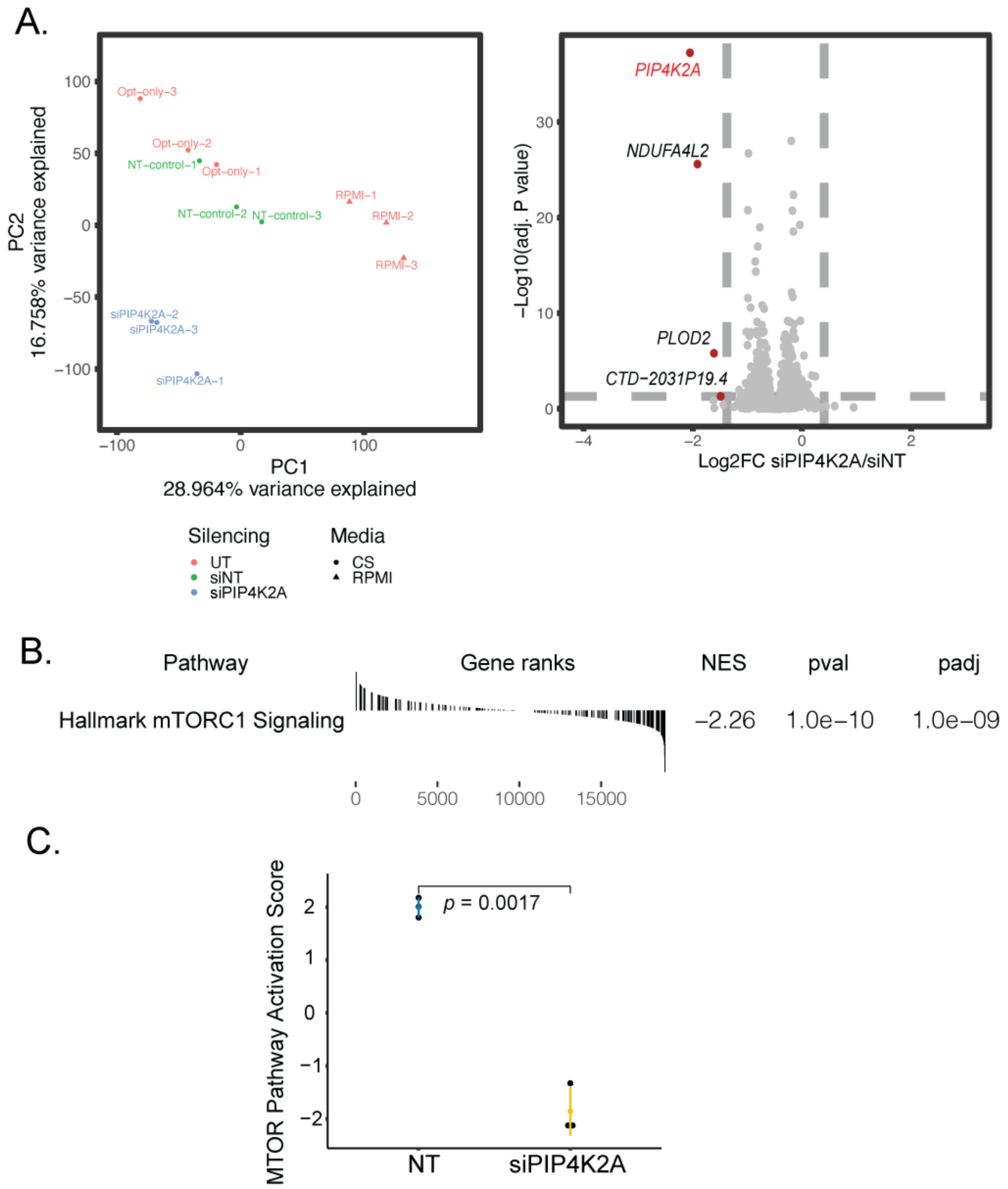

Figure S4. RNA Sequencing comparing NT controls with *PIP4K2A* knock down. (A) Significantly altered transcripts between treatment with siRNA targeting *PIP4K2A* (siPIP4K2A) and NT control treatments in AD conditions. Replicates distribution represented in principle component analysis. **(B)** Gene set enrichment for significant changes in mTORC1 Hallmark Signaling with siPIP4K2A knock down in AD conditions. **(C)** LNCaP RNA Sequencing data from siPIP4K2A in AD medium shows decrease in mTOR activation signature (p = 0.0017). t test values: n.s., not significant (p> 0.05), * p <0.05, ** p <0.01, *** p < 0.001.

Figure S5.

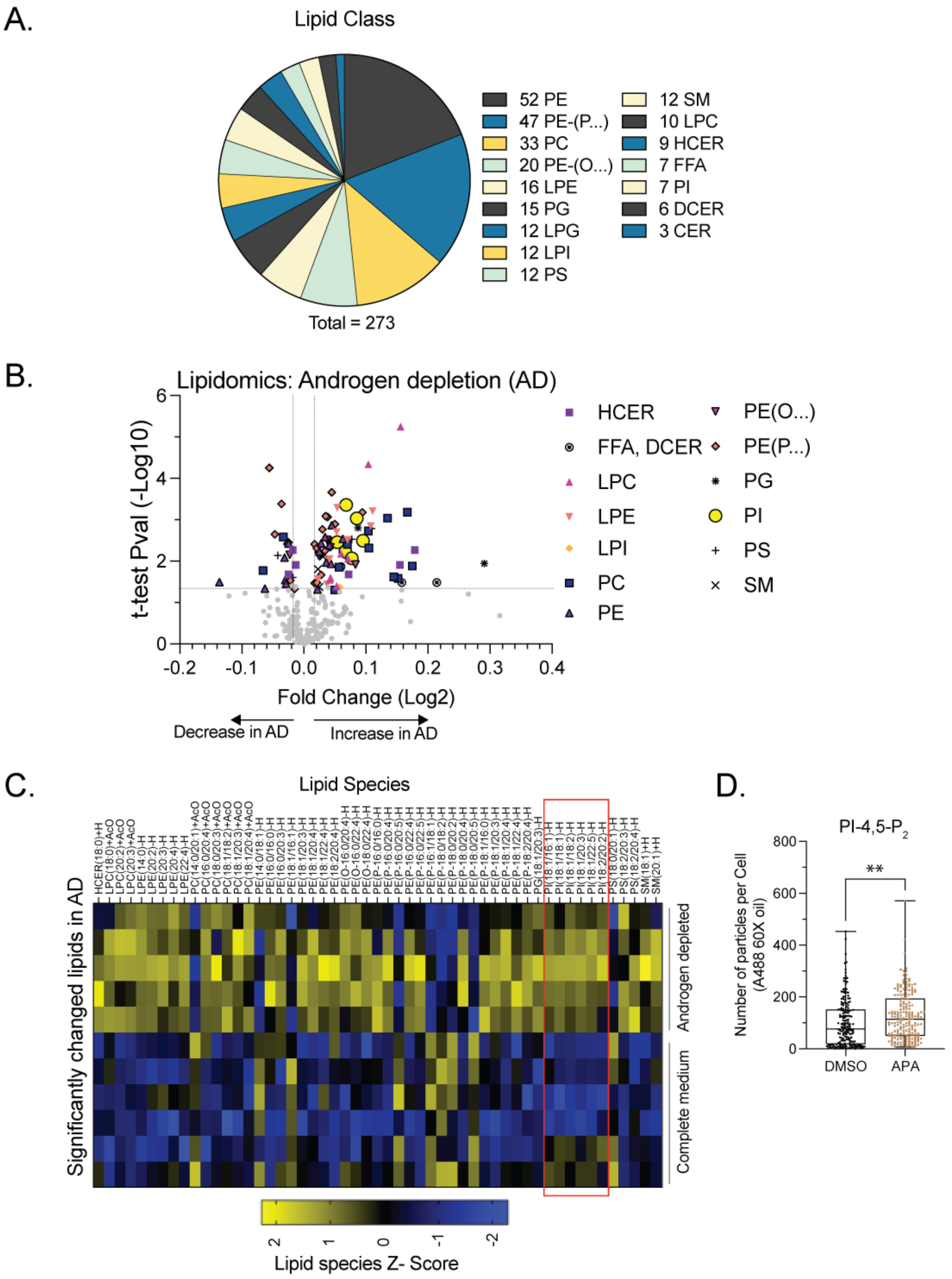
A.
Lipid Class
52 PE
47 PE-(P...)
33 PC
20 PE-(O...)
16 LPE
15 PG
12 LPG
12 LPI
12 PS
12 SM
10 LPC
9 HCER
7 FFA
7 PI
6 DCER
3 CER
Total = 273
B.
Lipidomics: Androgen depletion (AD)
t-test Pval (-Log10)
Fold Change (Log2)
Decrease in AD
Increase in AD
HCER
FFA, DCER
LPC
LPE
LPI
PC
PE
PE(O...)
PE(P...)
PG
PI
PS
SM
C.
Lipid Species
Significantly changed lipids in AD
Androgen depleted
Complete medium
Lipid species Z- Score
D.
PI-4,5-P2
Number of particles per Cell (A488 60X oil)
**
DMSO
APA

Figure S5. Lipidomics suggests changes in PI regulation. Lipidomics analysis of LNCaP cells in androgen deprivation (AD) conditions compared to RPMI complete medium. As well testing siRNA targeted *PIP4K2A* (siPIP4K2A) knock down compared to NT control. **(A)** Pie chart representing total 273 lipids detected within indicated lipid class indicated by pie slice color. **(B)** Volcano plot of significantly different lipid metabolites between complete RPMI medium and AD cultured LNCaP cells. Dot colors represent lipid class of individual lipid species. A threshold of $p < 0.05$ and fold change of at least ±0.1 fold change is used. Grey dots indicate lipids below thresholds and significance cutoffs. **(C)** Heatmap summarizing lipidomics Z scores of 53 significantly different lipid species between LNCaP cultured in complete RPMI +5% FBS compared to AD. Phosphatiylinositol (PI) lipids are highlighted (red box). **(D)** Intracellular pools of PI-4,5-P_2 are detected by immunofluorescent staining and number of PI-4,5-P_2 particles detected by confocal microscopy to compare PI-4,5-P_2 particles in LNCaP between 48hr treatment of 10μM APA and DMSO control. *t* test values: n.s., not significant ($p > 0.05$), * $p < 0.05$, ** $p < 0.01$, *** $p < 0.001$.

Figure S6.

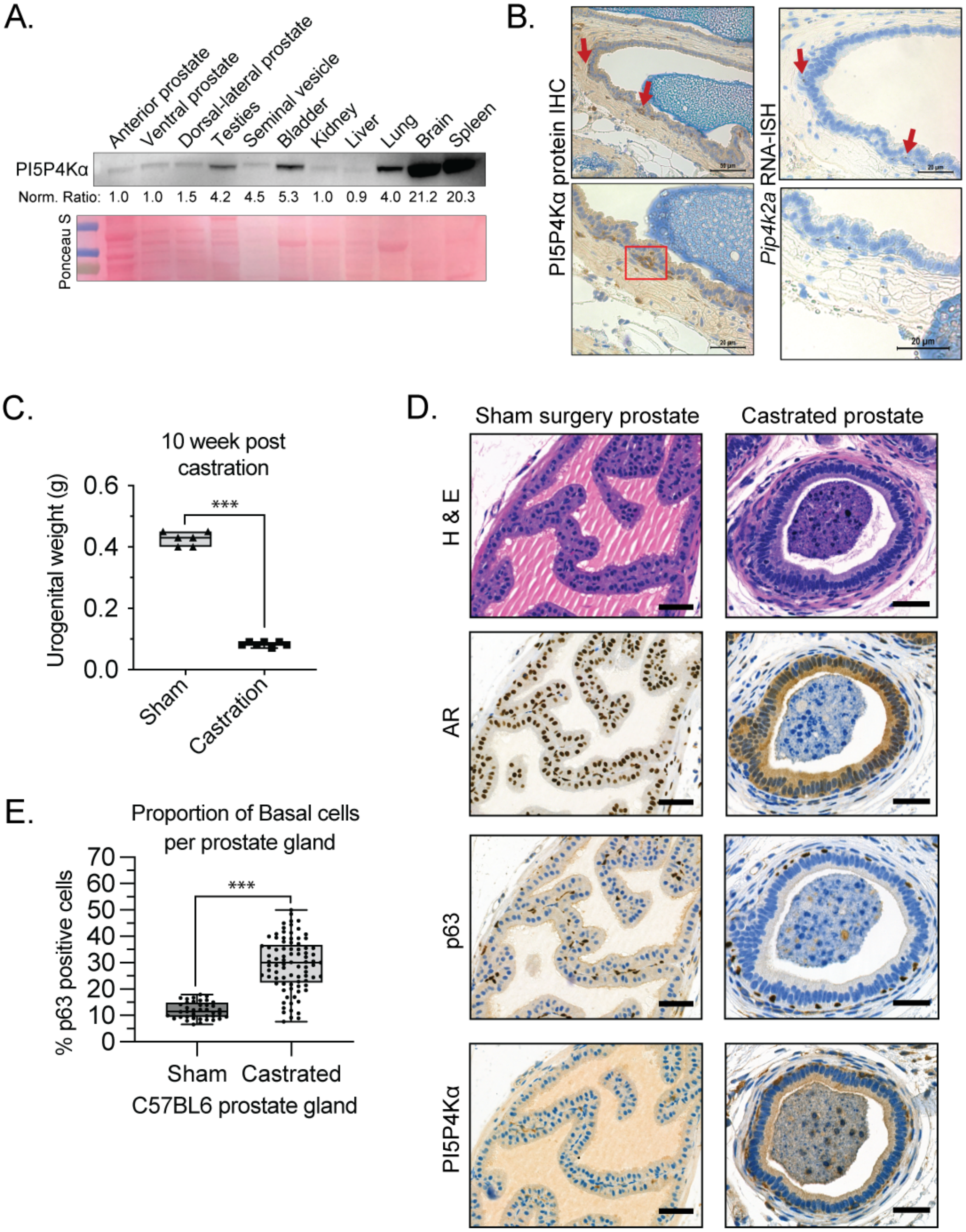

Figure S6. PI5P4Kα in mouse prostate tissue both normal adult and castrated. (A) Western blot analysis of protein expression of PI5P4Kα in mouse prostate lobes and a panel of various homogenized tissues. Protein stain Ponceau S is used as a general loading control. **(B)** Light phase imaging of tissue staining of normal

adult mouse anterior lobe. Serial section staining of PI5P4Kα protein antibody and RNA-ISH shows positive staining in basal cell population (red arrows). **(C)** C57BL/6J mice were aged to 8 weeks, surgically castrated or used for control sham surgery procedure. Tissues were harvested 10 weeks after surgery. Urogenital weight for castrated prostate compared to sham surgery animals is represented relative to animal bodyweight at time of necropsy (19.4% of sham, p =4.76E-13). **(D)** Representative light phase images for H&E, p63 (basal cell marker), AR (luminal cell marker), and PI5P4Kα IHC are shown for sham surgery and castrated prostate glands (scale bars = 20µm). **(E)** Quantification of p63 basal marker between sham and castrated tissue shows change in basal cell proportion in the castrated setting (t test, p = 5.03E-19).

Figure S7.

A.

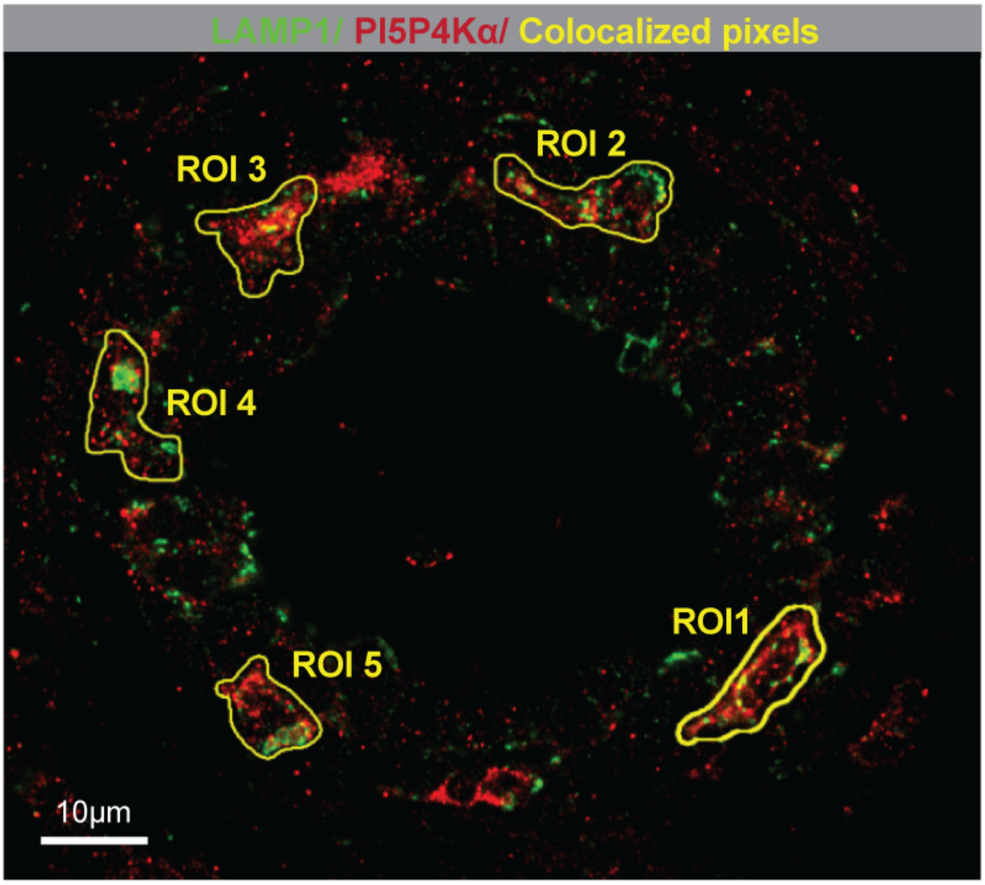

B.

Region of Interest	Pearson's Correlation (r)	Mander's Coefficients (with threshold) M1	Mander's Coefficients (with threshold) M2
1	0.538	0.711	0.254
2	0.397	0.426	0.271
3	0.528	0.754	0.175
4	0.282	0.323	0.242
5	0.502	0.578	0.251

A= Green channel (LAMP1)
B= Red channel (PI5P4Kα)

*M1 = fraction of A overlapping B
M2 = fraction of B overlapping A

C.

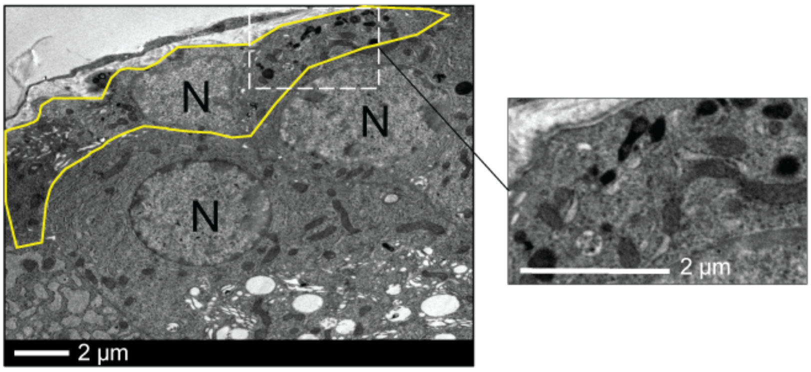

Figure S7. Lysosome imaging in mouse tissue. (A) Immunofluorescent staining of normal adult mouse prostate for colocalization of endo-lysosome marker, LAMP1 (green), and PI5P4Kα (red). Regions of interest (ROI) represent colocalization as calculated by Zeiss confocal microscope software. **(B)** Quantification of LAMP1 and PI5P4Kα colocalization using indicated regions of interest (ROIs). **(C)** Transmission electron microscope (TEM) imaging of normal adult mouse prostate. Yellow line outlines prostate basal cell, N = cell nucleus, white dashed box = cytoplasmic region of lysosome organelles.

Figure S8.

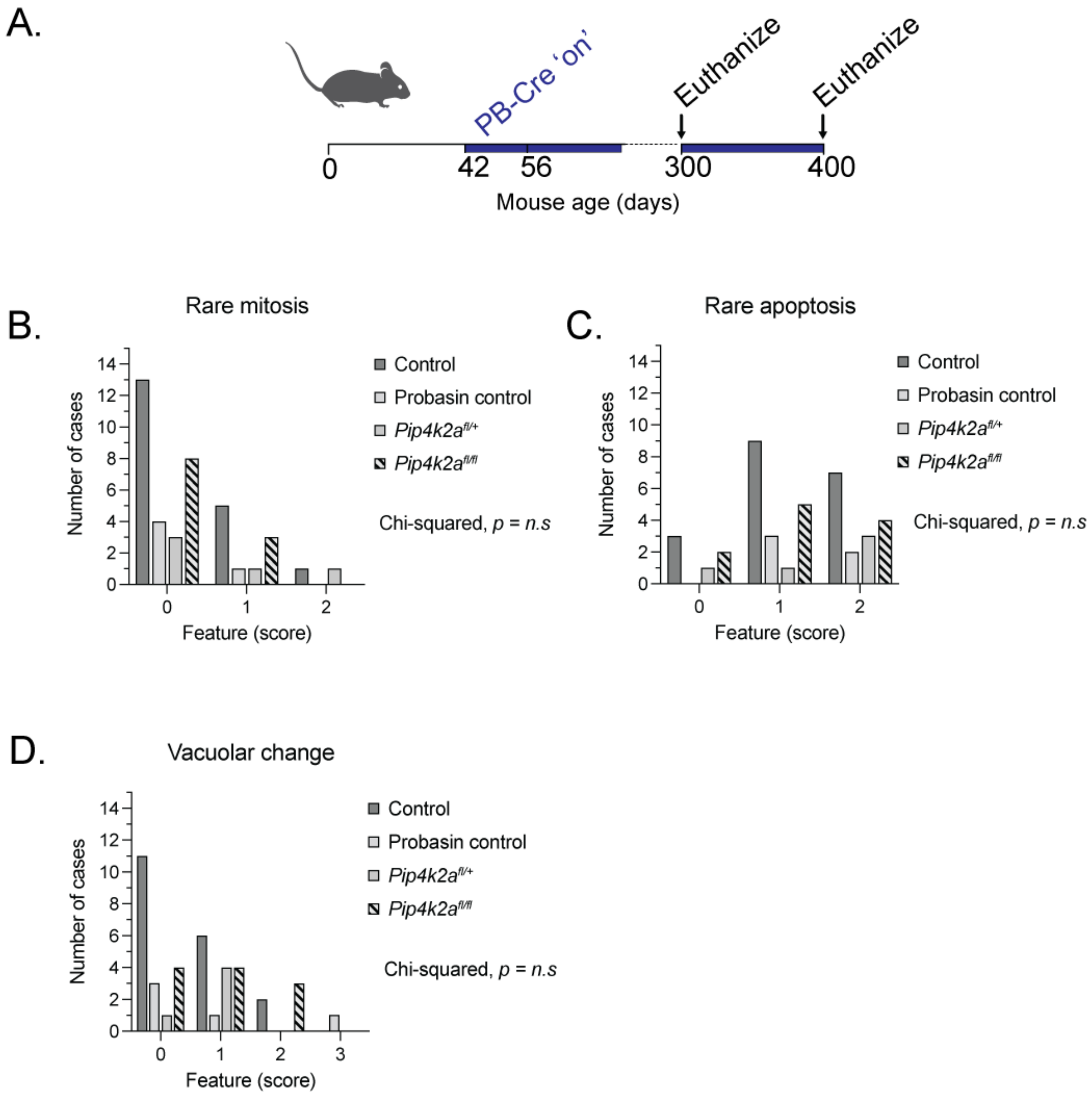

Figure S8. Prostate-specific genetically engineered mouse model. (A) Probasin-driven Cre recombinase system is used to delete *Pip4k2a*$^{fl/fl}$ alleles at time of mouse sexual maturity. Animals were aged over 300 and 400 days and tissues collected and evaluated by veterinary pathologist. Features of interested included **(B)** rare mitosis, **(C)** rare individual cell apoptosis, and **(D)** multifocal vacuolar cytoplasmic changes. Quantification demonstrated non-significant occurrence of features across genetic groups.

Figure S9.

A.

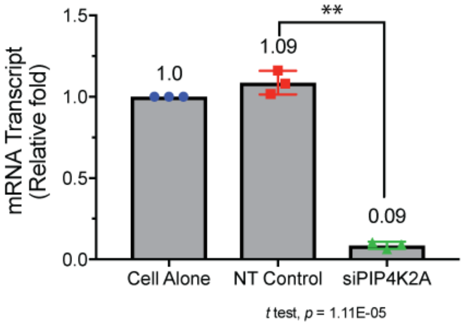

B.

	Untreated	NT control	siPIP4K2A
RPMI+5%FBS	67.5	68.3	85.7
48hr AD	83.1	82.2	93.7

C.

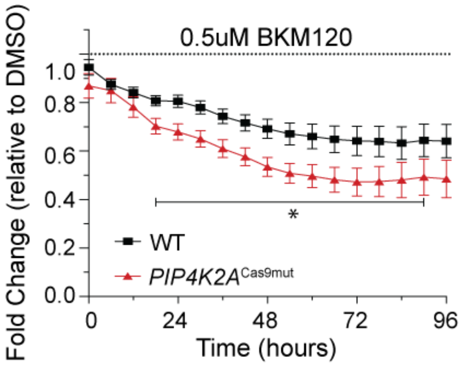

D.

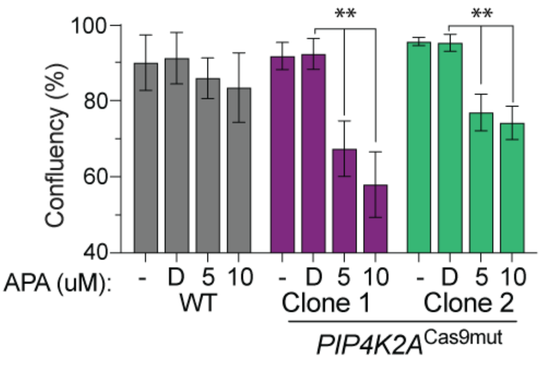

Figure S9. Targeting *PIP4K2A* increases cell stress. LNCaP cells were evaluated for changes in cell stress phenotypes following transfection with siRNA targeting *PIP4K2A* (siPIP4K2A) for 24hrs and an additional 48hrs of androgen deprivation (AD) medium. **(A)** Transcript knock down was confirmed with qRT-PCR (91% knock down; *t* test, p = 1.11E-05). **(B)** Knock down of siPIP4K2A produced increase in G1 phase cell population compared to control (G1: siPIP4K2A 85.7%, NT 68.3%) as measured propidium iodine flow cytometry with the greatest increase in AD medium (G1: *siPIP4K2A* 93.7%, NT 82.2%). **(C)** LNCaP *PIP4K2A*^{Cas9mut} cells experience greater sensitivity to PI3K inhibition with 0.5μM BKM120 treatment compared to wild-type (WT) control lines. **(D)** Mutant clones (*PIP4K2A*^{Cas9mut}) are also more sensitive to proliferation inhibition of APA in 5 and 10μM treatments for 144 hours. Data representative of 3 independent experiments mean ± SEM. *t* test values: n.s., not significant (p> 0.05), * p <0.05, ** p <0.01, *** p < 0.001.

Supplemental Tables

Supplemental Table S1. Genes involved in Androgen Receptor signature score for patient and organoid data sets corresponding to Fig. 1B, C, G.

gene_name	other_gene_name	gene_id	Source
PSA	*KLK3*	ENSG00000142515	Hieronymus et al. (ref 28)
TMPRSS2		ENSG00000184012	
NKX3-1		ENSG00000167034	
KLK2		ENSG00000167751	
GNMT		ENSG00000124713	
TMEPA1	*PMEPA1*	ENSG00000124225	
MPHOSPH9		ENSG00000051825	
ZBTB10		ENSG00000205189	
EAF2		ENSG00000145088	
BM039	*CENPN*	ENSG00000166451	
SARG	*C1orf116*	ENSG00000182795	
ACSL3		ENSG00000123983	
PTGER4		ENSG00000171522	
ABCC4		ENSG00000125257	
NNMT		ENSG00000166741	
ADAM7		ENSG00000069206	
FKBP5		ENSG00000096060	
ELL2		ENSG00000118985	
MED28		ENSG00000118579	
HERC3		ENSG00000138641	
MAF		ENSG00000178573	
TNK1		ENSG00000174292	
GLRA2		ENSG00000101958	
MAPRE2		ENSG00000166974	
PIP5K2B		ENSG00000276293	
MAN1A1		ENSG00000111885	
CD200		ENSG00000091972	

Supplemental Table S2. 3D organoid characteristics and AR signature score corresponding to Fig. 1G.

Gene expression from bulk RNA Sequencing in FPKM

3D Organoid	Tumor Characterization	AR Score	Source
MSKPCa1	NEPC	0.25363087	Gao et al. (ref 32), Tang et al. (ref 33)
MSKPCa2	adenocarcinoma	0.49100811	
MSKPCa3	adenocarcinoma	0.30191469	
MSKPCa4	NEPC	0.20583733	
MSKPCa5	adenocarcinoma	0.54667684	
MSKPCa6	adenocarcinoma	0.29564061	
MSKPCa7	adenocarcinoma	0.39702358	
MSKPCa8	adenocarcinoma	0.43477918	
MSKPCa9	adenocarcinoma	0.47695057	
MSKPCa10	NEPC	0.14922818	
MSKPCa11	adenocarcinoma	0.4636689	
MSKPCa12	adenocarcinoma	0.30080169	
MSKPCa13	adenocarcinoma	0.38834034	
MSKPCa14	NEPC	0.1890031	
MSKPCa15	adenocarcinoma	0.27613832	
MSKPCa16	NEPC	0.17309722	
MSKPCa17	adenocarcinoma	0.33381262	
WCMC_PM155	NEPC	0.20323665	Puca et al. (ref 31), Tang et al. (ref 33)
WCMC_PM154	NEPC	0.02049771	Puca et al. (ref 31), Tang et al. (ref 33)

NEPC: neuoendocrine prostate cancer

Supplemental Table S3. Transcript data for PIP4K2 isoforms in PCa organoid lines corresponding to Fig. 1G.

Gene expression from bulk RNA Sequencing in FPKM

gene_id	ENSG00000150867	ENSG00000141720	ENSG00000166908
gene_name	***PIP4K2A***	***PIP4K2B***	***PIP4K2C***
MSKPCa1	6.90387	33.2673	41.3038
MSKPCa2	5.08179	16.9063	21.5695
MSKPCa3	7.68078	27.3704	40.1624
MSKPCa4	22.9569	42.0516	20.631
MSKPCa5	3.21198	17.6167	41.6427
MSKPCa6	3.06547	14.1237	32.1438
MSKPCa7	6.79925	18.7924	40.2106
MSKPCa8	16.3264	13.1729	51.2169
MSKPCa9	13.0404	22.0357	39.2833
MSKPCa10	6.89783	28.9361	23.5565
MSKPCa11	6.96129	25.356	30.5817
MSKPCa12	17.8058	12.3567	48.0065
MSKPCa13	4.29868	16.5135	29.4029
MSKPCa14	9.69569	24.1764	44.7784
MSKPCa15	8.38858	39.4781	24.9718
MSKPCa16	15.3647	20.4909	82.9227
MSKPCa17	11.3069	11.2192	36.5931
WCMC_PCa7	6.57316	12.9895	40.4021
WCMC_PM154	23.2393	20.7928	110.31

Supplemental Table S4. Reagents used

Reagent	Company	Catalog No.
Fetal Bovine Serum, charcoal stripped	Thermo Fisher Scientific	A3382101
ON-TARGETplus Human PIP4K2A (5305) siRNA - SMARTpool	Dharmacon	L-006778-00-0005
ON-TARGETplus Non-targeting Pool	Dharmacon	D-001810-10-05
Apalutamide (APA; ARN-509)	Selleck Chemicals	S2840
Rapamycin (Sirolimus)	Selleck Chemicals	S1039
Buparlisib (BKM120)	Selleck Chemicals	S2247
2-Deoxy-D-glucose	Sigma-Aldrich	D8375
Senescence β-Galactosidase Staining Kit	Cell Signaling	9860S
5α-Androstan-17β-ol-3-on (DHT)	Sigma-Aldrich	10300
LysoTracker Deep Red	Invitrogen	L12492

Supplemental Table S5. Antibodies used

Antibody	Company	Catalog No.	Purpose	Conditions
PIP4K2A (D83C1) Rabbit mAb	Cell Signaling	5527S	Western blot	1:1000 in 5% Milk
Anti-PIP4K2A Rabbit Polyclonal Antibody	ProteinTech	12469-1-AP	IHC	1:200, HIER TRIS
Anti-PIP2 antibody [2C11]	Abcam	Ab11039	IF	Hammond et al. (ref 48)
Beta-Actin [AC-15]	Abcam	Ab6276	Western blot	1:5000 in 5% BSA
AR [EPR1535(2)]	Abcam	Ab133273	Western blot	1: 1000 in 5% BSA
Ar	Dako-Agilent	M3562	IHC	1:100, HIER TRIS
NKX3.1	Cell Signaling	83700	Western blot	1: 1000 in 5% BSA
KLK3/PSA (D6B1)	Cell Signaling	5365	Western blot	1: 1000 in 5% BSA
Anti-LAMP1 antibody [1D4B]	Abcam	Ab24871	IHC	1:200, HIER TRIS, 48hr incubation
p63	Biosystems	NCL-L-p63	IHC	1:200, HIER TRIS
GFP Antibody [DyLight 488]	Novus Biologicals	NBP1-69969	IHC	1:200, HIER TRIS
Phospho-AKTS473	Cell Signaling	4060	Western blot	1: 1000 in 5% BSA
Pan AKT (C67E7)	Cell Signaling	4691	Western blot	1: 1000 in 5% BSA
Phospho-S6 Ribosomal Protein (Ser235/236)	Cell Signaling	2211	Western blot	1: 1000 in 5% BSA
S6 Ribosomal Protein (5G10)	Cell Signaling	2217	Western blot	1: 1000 in 5% BSA
Phospho-4E-BP1 (Thr70)	Cell Signaling	9455	Western blot	1: 1000 in 5% BSA
4E-BP1 (53H11)	Cell Signaling	9644	Western blot	1: 1000 in 5% BSA
Alexa Fluor® 488-AffiniPure Rabbit Anti-Goat IgG (H+L)	Jackson ImmunoResearch	305-545-003	IF	1:300 in Goat serum
Cy3-AffiniPure Goat Anti-Rabbit IgG (H+L)	Jackson ImmunoResearch	111-165-003	IF	1:300 in Goat serum

Supplemental Table S6. Primer sequences

Transcript	Species	Forward sequence (5'-3')	Reverse sequence (5'-3')
ACTB	Human	GCGAGAAGATGACCCAGATC	CCAGTGGTACGGCCAGAGG
GAPDH	Human	CCAGGTGGTCTCCTCTGACTTC	TCATACCAGGAAATGAGCTTGACA
PIP4K2A	Human	GGGGGTAAACCACTCGATCA	ATTCCAAACCTCTCCCGCAG
PIP4K2B	Human	GAAGCATTTCGTGTGCCAGA	GCTGGGCAGGTTCTCCTTAT
PIP4K2C	Human	CAGGGAAAATCTGCCCAGTC	CAGGGAAAATCTGCCCAGTC
AR	Human	CCAGGGACCATGTTTTGCC	CCAGGGACCATGTTTTGCC
KLK3	Human	GTTGTCTTCCTCACCCTGTCC	GGTTGGGAATGCTTCTCG
FKBP5	Human	GCAACAGTAGAAATCCACCTG	CTCCAGAGCTTTGTCAATTCC
TMPRSS2	Human	GGACAGTGTGCACCTCAAAGAC	TCCCACGAGGAAGGTCCC
OPRK1	Human	AACTCGCTGGTCATGTTCGT	CTCTGAAAGGGCATGGTTGT
Actb	Mouse	TACCACCATGTACCCAGGCA	CTCAGGAGGAGCAATGATCTTGAT
Pip4k2a	Mouse	GTGGCAGAGATGCACAACATC	CCGCAGGTTACGGAAGACCATC
Ar	Mouse	GCCAGGAGTGGTGTGTGCCG	AAGTTGCGGAAGCCAGGCAAGG
Nkx3.1	Mouse	ATGCTTAGGGTAGCGGAG	TGCGGATTGCCTGAGTGTC
Fkbp5	Mouse	TGAGGGCACCAGTAACAATGG	CAACATCCCTTTGTAGTGGACAT

Supplemental Material as Excel files:

Supplemental Data S1. RNA Sequencing gene counts and pathway analysis.

Supplemental Data S2. Lipidomics analysis data.

Supplemental Data S3. Western blot source data.
